# Predicting *Plasmodium knowlesi* transmission risk across Peninsular Malaysia using machine learning-based ecological niche modeling approaches

**DOI:** 10.3389/fmicb.2023.1126418

**Published:** 2023-02-16

**Authors:** Wei Kit Phang, Mohd Hafizi bin Abdul Hamid, Jenarun Jelip, Rose Nani binti Mudin, Ting-Wu Chuang, Yee Ling Lau, Mun Yik Fong

**Affiliations:** ^1^Department of Parasitology, Faculty of Medicine, Universiti Malaya, Kuala Lumpur, Malaysia; ^2^Disease Control Division, Ministry of Health Malaysia, Putrajaya, Malaysia; ^3^Sabah State Health Department, Ministry of Health Malaysia, Kota Kinabalu, Sabah, Malaysia; ^4^Department of Molecular Parasitology and Tropical Diseases, School of Medicine, College of Medicine, Taipei Medical University, Taipei, Taiwan

**Keywords:** *Plasmodium knowlesi*, Peninsular Malaysia, ecological niche modeling, XGBoost, ensemble modeling, maximum entropy

## Abstract

The emergence of potentially life-threatening zoonotic malaria caused by *Plasmodium knowlesi* nearly two decades ago has continued to challenge Malaysia healthcare. With a total of 376 *P. knowlesi* infections notified in 2008, the number increased to 2,609 cases in 2020 nationwide. Numerous studies have been conducted in Malaysian Borneo to determine the association between environmental factors and knowlesi malaria transmission. However, there is still a lack of understanding of the environmental influence on knowlesi malaria transmission in Peninsular Malaysia. Therefore, our study aimed to investigate the ecological distribution of human *P. knowlesi* malaria in relation to environmental factors in Peninsular Malaysia. A total of 2,873 records of human *P. knowlesi* infections in Peninsular Malaysia from 1st January 2011 to 31st December 2019 were collated from the Ministry of Health Malaysia and geolocated. Three machine learning-based models, maximum entropy (MaxEnt), extreme gradient boosting (XGBoost), and ensemble modeling approach, were applied to predict the spatial variation of *P. knowlesi* disease risk. Multiple environmental parameters including climate factors, landscape characteristics, and anthropogenic factors were included as predictors in both predictive models. Subsequently, an ensemble model was developed based on the output of both MaxEnt and XGBoost. Comparison between models indicated that the XGBoost has higher performance as compared to MaxEnt and ensemble model, with AUC_ROC_ values of 0.933 ± 0.002 and 0.854 ± 0.007 for train and test datasets, respectively. Key environmental covariates affecting human *P. knowlesi* occurrence were distance to the coastline, elevation, tree cover, annual precipitation, tree loss, and distance to the forest. Our models indicated that the disease risk areas were mainly distributed in low elevation (75–345 m above mean sea level) areas along the Titiwangsa mountain range and inland central-northern region of Peninsular Malaysia. The high-resolution risk map of human knowlesi malaria constructed in this study can be further utilized for multi-pronged interventions targeting community at-risk, macaque populations, and mosquito vectors.

## 1. Introduction

Environmental variations including land cover types, climate changes, anthropogenic landscapes, and host distributions have been linked to the geographical distribution and altered transmission patterns of malaria and other vector-borne diseases worldwide (Medone et al., 2015; Morand and Lajaunie, 2021; Kulkarni et al., 2022). In Malaysia, the transmission of the simian malaria species *Plasmodium knowlesi*, *via Anopheles* Leucosphyrus group mosquitoes, has been attributed to environmental changes affecting the proximity between people, macaque reservoirs (mainly *Macaca fascicularis* and *M. nemestrina*), and mosquito vectors (Cuenca et al., 2021). It is important to highlight that the incidence of human knowlesi malaria has grown significantly over the last two decades, threatening the malaria elimination efforts in Malaysia and other Southeast Asian countries (Singh et al., 2004; Shearer et al., 2016; Chin et al., 2020). It is suggested that the increasing reports of human knowlesi malaria are driven by deforestation, agricultural expansion, and spatial overlaps between the human population and wildlife hosts (Moyes et al., 2016; Fornace et al., 2019).

Malaysia is geographically divided by the South China Sea into two regions, Peninsular Malaysia and Malaysian Borneo. Heterogeneities exist in the distribution of *P. knowlesi* vectors between these regions such as *An. cracens*, *An. introlatus*, and *An. hackeri* in Peninsular Malaysia, and *An. balabacensis* and *An. latens* in Malaysian Borneo (Tan et al., 2008; Wong et al., 2015; Ang et al., 2020; Jeyaprakasam et al., 2021a). Molecular epidemiological studies have found that the geographical separation could have also driven the allopatric divergence of *P. knowlesi* into distinct subpopulations (Divis et al., 2017). Studies in Sabah, a state in Malaysian Borneo, have demonstrated the association between environmental factors and knowlesi malaria risk (Brock et al., 2019; Fornace et al., 2019; Sato et al., 2019; Hod et al., 2022). However, environmental influences on knowlesi malaria in Peninsular Malaysia are not widely studied. Therefore, it is of interest to know how environmental factors may impact knowlesi malaria transmission in Peninsular Malaysia.

As a part of the malaria intervention strategy in Malaysia, disease screening *via* active case detection, mass blood survey, and entomological surveillance were conducted mainly in localities with a history of malaria cases. This intervention strategy is not able to effectively cover other parts of the populations which are at high-risk or may be exposed to the disease without case notifications, especially among Orang Asli (i.e., indigenous people) communities in forested areas lacking accessible roads. Also, not knowing the locations of the high-risk area may affect the systematic implementation of macaque reservoir screening and entomological surveillance. Therefore, identifying the ecological niche of the disease can support plans for controlling disease transmission.

The emerging role of machine learning approaches in healthcare and spatial epidemiology is instrumental, especially in modeling the covariate contribution toward disease transmission as well as to predict the spatial distribution of the disease (Kopczewska, 2022; Temenos et al., 2022). For instance, MaxEnt (maximum entropy) algorithm enables the estimation of the geographical range of a target disease by determining the probability distribution of maximum entropy (i.e., most spread out or closest to uniform) based on the availability of case presence and ecological information within the study area (Phillips et al., 2006). Besides, decision-tree-based models such as random forest and gradient-boosted tree are popularly used in ecological niche modeling. These models have been widely applied to estimate the potential risk areas of diseases such as malaria (Bhatt et al., 2017), dengue (Liu et al., 2016), West Nile virus (Shartova et al., 2022), scrub typhus (Acharya et al., 2019), brucellosis (Jia and Joyner, 2015), and Chagas disease (Mischler et al., 2012) as well as to estimate the spatial distribution of the vectors of Lyme disease (Burrows et al., 2022), chikungunya (Richman et al., 2018), leishmaniasis (Cunze et al., 2019), and malaria (Akpan et al., 2018). Previous studies have demonstrated the use of boosted regression tree (BRT) to map the geographical distribution of natural reservoirs and vectors of *P. knowlesi* and estimated the risk of *P. knowlesi* infection throughout Southeast Asia (Moyes et al., 2016; Shearer et al., 2016). Also, several studies applied ensemble modeling techniques by integrating multiple predictive models to generate a prediction of malaria risk with higher performance (Bhatt et al., 2017; Chemison et al., 2021).

A relatively new approach known as extreme gradient boosting (XGBoost), was found to outperform various models in spatial modeling (Zhao et al., 2021). In addition to improving the model performance, understanding the influence of each parameter in the model is important for public health administration. Recently, SHAP (SHapley Additive exPlanations) tool has rendered detailed explanations to once-considered black-box machine learning models without sacrificing performance. This approach is coupled with XGBoost as a method emphasized in this study.

Understanding the transmission patterns and geographical distribution of *P. knowlesi* in Peninsular Malaysia is essential to strategize effective disease control measures and enhance understanding of how ecologies affect the risks of knowlesi malaria. To address these needs, we aimed to investigate the impacts of diverse environmental variations toward human knowlesi malaria occurrence as well as to predict potential high-risk areas for human knowlesi malaria at fine spatial resolution across Peninsular Malaysia using machine learning models of MaxEnt and XGBoost.

## 2. Materials and methods

### 2.1. Ethic statement

This study was registered with the National Medical Research Register (NMRR-16-2,109–32,928), and ethical approval was obtained from the Malaysian Research Ethical Committee (MREC) [reference no. KKM/NIHSEC/P16-1782 (11)]. For all case data, information that identifies the patient was anonymized.

### 2.2. Geography of Peninsular Malaysia

**Figure 1:**
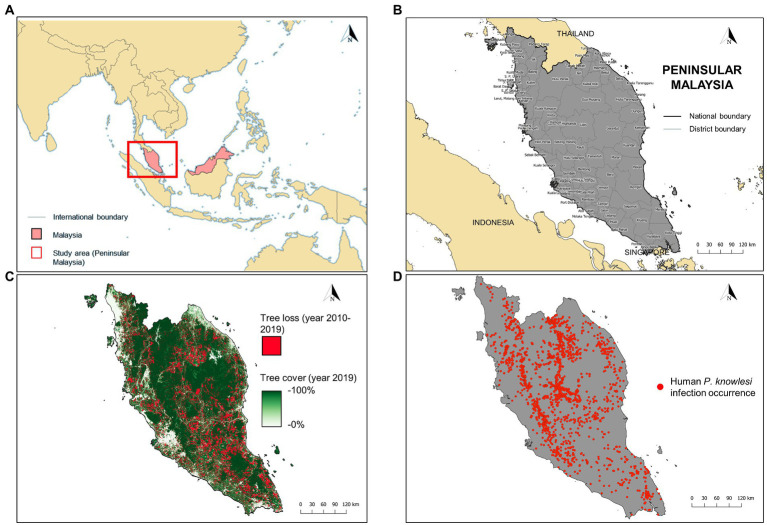
**(A)** Location of Peninsular Malaysia (red box). **(B)** The district-level administrative boundary of Peninsular Malaysia. **(C)** Extent of tree loss (recorded in years 2010–2019) and tree cover (recorded in year 2019) in Peninsular Malaysia. Tree loss data was acquired from Global Forest Change database (https://earthenginepartners.appspot.com/science-2013-global-forest/download_v1.7.html) whereas tree cover data was acquired from Copernicus Global Land Service (https://zenodo.org/record/3939050#.Yw-ZpXZBzIU). **(D)** Geolocated cases of human knowlesi malaria (*n* = 2,873) throughout Peninsular Malaysia from years 2011–2019.

Malaysia is a country in Southeast Asia and has two regions, Peninsular Malaysia and Malaysian Borneo (Figure 1A). Our study focused on Peninsular Malaysia which extends from latitude 1°15′50.0″N to 6°43′36.0″N and from longitude 99°35′E to 104°35″E (Figure 1B). From 2010 to 2019, Peninsular Malaysia experienced a loss of 2.26 million hectares of tree cover (Global Forest Watch, 2021; Figure 1C). Within this period, at least 90% of the tree loss was attributable to deforestation activities (Global Forest Watch, 2021). Previous studies suggested that landscape changes driven by deforestation would increase the likelihood of spillover of the macaque population into the human population, thus, increasing the risk of knowlesi malaria exposure (Fornace et al., 2016).

### 2.3. Human knowlesi malaria data

**Figure 2:**
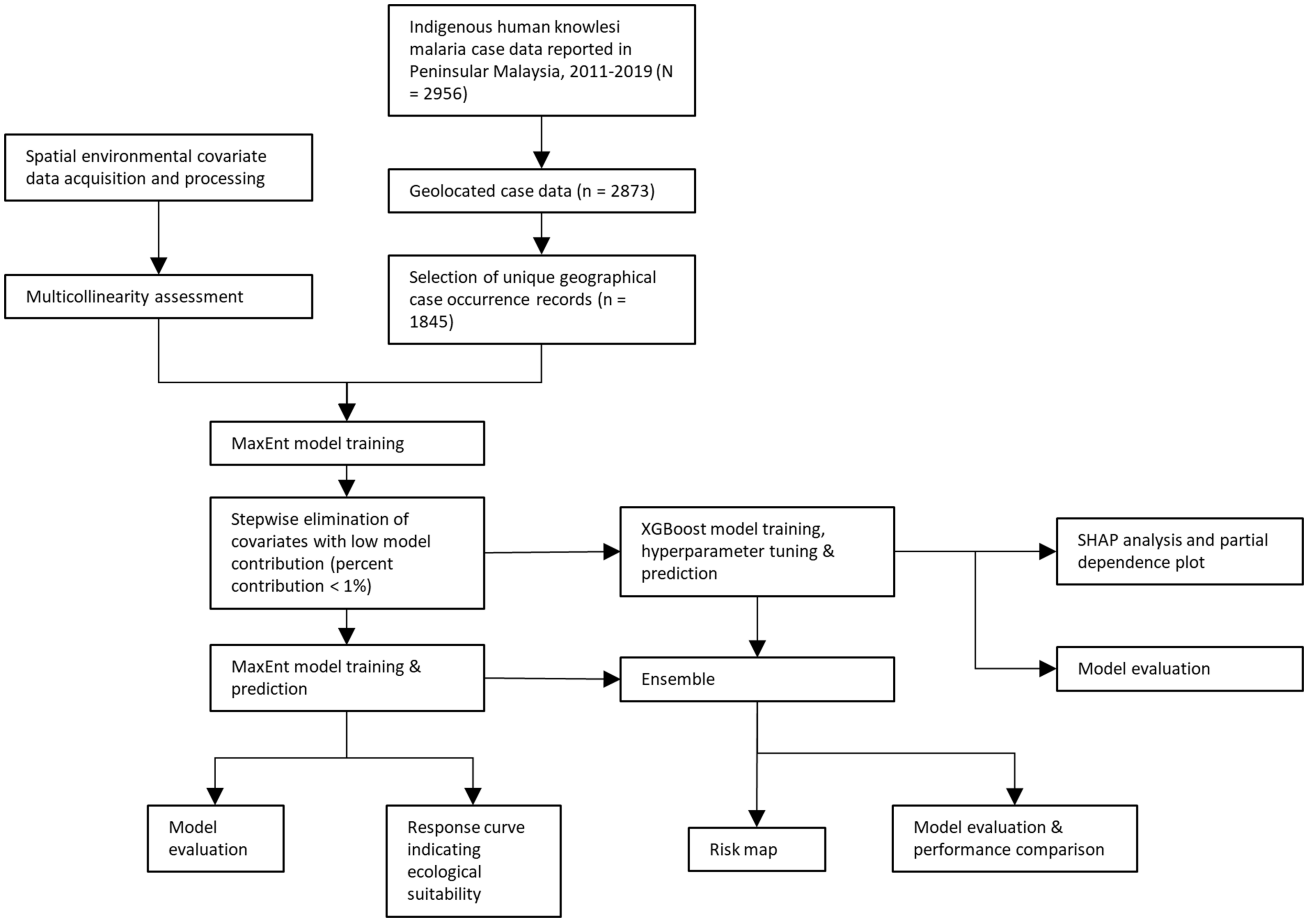
Schematic summarizing the modeling procedure in this study.

In Malaysia, all laboratory-diagnosed malaria cases are notified to the District Health Offices and State Health Departments, which will be subsequently compiled by the Ministry of Health Malaysia. Human knowlesi malaria cases are diagnosed *via* microscopic examination and/or nested PCR assay. In this study, retrospective data on knowlesi malaria cases from 1st January 2011 to 31st December 2019 were provided by the Ministry of Health Malaysia. Approximately 97.16% (*n* = 2,873) of the reported indigenous knowlesi malaria cases (total = 2,956) were able to be geolocated (Figure 1D). The source of infection reported for each case was manually geolocated as the occurrence point with reference to Google Maps (Google, 2022), Mapcarta (Mapcarta, 2022), Waze (Waze Mobile, 2022), as well as state and federal territory gazetteers (Ministry of Energy and Natural Resources Malaysia, 2022). For cases with no information on the source of infection addresses, household or working addresses were used as the replacement for occurrence point (9.89%, *n* = 284, of the geolocated cases were georeferenced this way). Before running MaxEnt and XGBoost modeling, reports of cases within the same grid in a covariate layer were considered as a single unique record. This approach was used to reduce spatial clumping and avoid the inflation of model accuracy (Veloz, 2009). Overall, the case dataset consisted of 1,845 unique occurrence records. The overview of the modeling procedure is shown in Figure 2.

### 2.4. Spatial environmental covariate data collation and processing

ArcGIS Pro version 2.7.2 (Esri, Redlands, CA, United States) and QGIS version 3.6.3 (Open Source Geospatial Foundation, Beaverton, OR, United States) were used to visualize and process all spatial data. Original covariate data were acquired from multiple sources and processed as described in Supplementary Data and Supplementary Tables 1–3. The coordinate reference systems of all spatial data were projected to World Geodetic System (WGS) 84/Universal Transverse Mercator (UTM) zone 47 N. All covariates were resampled to produce raster layers with 1×1 km^2^ pixel spatial resolution. A total of 36 constructed covariate spatial data consisted of landscape, climate, anthropogenic, and proximity characteristics were used for subsequent analysis (Supplementary Figures 1–3).

### 2.5. Multicollinearity test

A multicollinearity assessment was conducted to remove highly correlated covariates *via* two steps (Sillero et al., 2021). Firstly, a pairwise correlation matrix was constructed and Pearson’s correlation coefficient *r* ≤ −0.8 or ≥ 0.8 were set as a threshold to selectively remove highly correlated covariates. Then, an assessment based on the variance inflation factor (VIF) was conducted to remove covariates with VIF ≥ 10.

### 2.6. Maximum entropy (MaxEnt) modeling procedure

MaxEnt is a machine learning approach which applies a maximum entropy algorithm to model potential distributions of an object based on presence-only datasets. MaxEnt version 3.4.4 (Phillips et al., 2006) was used in this study to construct the presence-background niche model for knowlesi malaria in Peninsular Malaysia. The unique case occurrence dataset was randomly partitioned into train dataset (70%) and test dataset (30%) through subsampling approach. Log-transformed value of human population density covariate was selected as the sampling bias layer. Sampling bias layer was included to account for the assumption of a greater likelihood of disease detection in populous places (Merow et al., 2013). The inclusion of sampling bias layer could also reduce the likelihood of false positives such as predicting highly populated areas as high-risk areas due to biased detection location. In this model, 10,000 background points were randomly sampled. The modeling software factors out bias by assigning weights to the background points based on the sampling bias layer value during modeling. The modeling parameters used include regularization multiplier of 1, 2000 iterations, and 0.00001 convergence threshold. The area under curve of receiver operating characteristic (AUC_ROC_) was used to evaluate the performance of the model. The higher the AUC_ROC_ value (ranging from 0 to 1), the higher its accuracy. The logistic output of the model was selected to present the predicted risk probability.

All environmental covariates (except human population density) that passed the multicollinearity assessment were included in the model training stage. Ten replicated models were fitted with each trained to a separate subsampled dataset. The relative importance of each covariate was ranked based on the percent contribution to the model. Backward stepwise elimination was applied to the to remove the covariates with the lowest percent contribution to the models until all remaining covariates have a percent contribution threshold of ≥1%.

To obtain a robust model, 30 replicated models were developed using the final covariate dataset (Convertino et al., 2012; Acharya et al., 2018). Mean output grids were calculated among the raster outputs of these 30 models and these grids were used to generate a 1×1 km^2^ pixel spatial resolution predicted risk map of human knowlesi malaria. Ecological suitability ranges of the human knowlesi malaria transmission per covariate were demonstrated by response curves.

### 2.7. Extreme gradient boosting (XGBoost) modeling procedure

XGBoost is a machine learning algorithm based on gradient boosting, which can be utilized for both regression and classification problems. XGBoost is known for its ability to speed up data learning execution out of core computation (Chen and Guestrin, 2016). Similar to MaxEnt, we employed XGBoost as a presence-only model by using the same dataset in the MaxEnt procedure, consisting of case occurrence and background points. This dataset was transformed into binary code of 1 and 0 to indicate case occurrence and background data, respectively. The covariates utilized for the final MaxEnt was similarly employed as predictors in XGBoost modeling. The partitioning of the case dataset into 70% train and 30% test datasets was the same as previously mentioned in the MaxEnt modeling procedure. We constructed the XGBoost model with a tree-based booster learning type and set the objective of binary logistic regression. It was noted that the background data make up a large proportion of the dataset by approximately five-fold as compared to the case occurrence data. This would lead to an imbalanced dataset, which can affect the model performance and cause biased prediction toward higher proportion class of background data. Therefore, we assigned a class weighted approach to reduce the impact of imbalanced data issue. The weight for each class (occurrence class weight, *w*_1_, and background class weight, *w_0_*) can be calculated as follows:


$$w_{1}=\frac{N_{\text{train}}}{2N_{\left(\text{train},1\right)}}$$

$$w_{0}=\frac{N_{\text{train}}}{2N_{\left(\text{train},0\right)}}$$

where *N_train_* is the total number of data points (both occurrence and background) in the train dataset, *N_(train,1)_* and *N_(train,0)_* are the numbers of occurrences and backgrounds, respectively, in train dataset. Weight assignment allows the handling of class imbalance by reducing model bias toward the majority class without manipulating the training data distribution (Johnson and Khoshgoftaar, 2019). Besides class weight, we included the bias layer of log-transformed human population density value as the instance weight for each corresponding occurrence and background points to adjust sampling bias. Class weight and instance weight were processed prior to input into the train dataset. AUC_ROC_ was used to evaluate the performance of the model. During model training process, hyperparameter tuning was conducted to identify optimal parameters while maximizing the model training AUC_ROC_. Five-fold cross-validation of the train dataset was performed during the tuning phase to avoid overfitting the model prediction. The final optimized parameters are described in Supplementary Table 4. Mean output grids were calculated among the raster outputs of 30 XGBoost replicates, and these grids were used to generate a 1×1 km^2^ pixel spatial resolution predicted risk map of human knowlesi malaria.

To provide better interpretations of environmental conditions and knowlesi malaria risk, we applied SHapley Additive exPlanations (SHAP) to disseminate and interpret the output of XGBoost model (Campbell et al., 2022). SHAP values were generated to evaluate the relative importance of covariates in the model. A high and positive SHAP value indicates that the covariate highly and positively affects the output of the prediction model and vice versa (Lundberg et al., 2020). Global SHAP summary plots and SHAP dependence plots were created to explain the relationship between covariates and the model prediction output. XGBoost modeling procedure was performed in R using maptools, raster, and usdm packages to manage digital mapping and data extraction, dplyr package for data manipulation, XGBoost package for running XGBoost algorithm, caret package for managing machine learning framework and hyperparameter tuning, pROC package for analyzing model AUC_ROC_, and SHAPforxgboost package for generating SHAP value and plots.

### 2.8. Ensemble model procedure

Ensemble modeling involves the aggregation of outcome prediction from multiple model algorithms to generate a final prediction. Model ensemble approach is frequently applied to address machine learning issues such as incremental learning, imbalanced data, error correction, and confidence estimation, and it usually generates improved results (Polikar, 2012). An ensemble model was developed by averaging the outputs of MaxEnt and XGBoost models using the same subsampled datasets as used for constructing both MaxEnt and XGBoost. The averaged ensemble output was used to generate human knowlesi malaria risk map. The predictive performances of MaxEnt, XGBoost, and ensemble models were evaluated using AUC_ROC_, sensitivity, specificity, and F1-score. To compare the prediction patterns produced by different models, 20,000 points were randomly sampled from the risk map outputs of the three models and converted by kernel density. District-level annual incidence rate in 1 million people was calculated by dividing the annual number of reported cases by estimated mid-year population size and multiplying by 1,000,000. Spearman’s correlation test was conducted to determine the correlation between variables with *value of p* <0.05 indicates statistical significance. The procedure of model development and validation was carried out in R software. The R script used to conduct XGBoost and ensemble modeling is available at https://github.com/WKPhang/XGBoost_EcologicalNicheModel/.

### 2.9. Identification of priority areas for intervention and surveillance

Priority zone maps were developed to identify priority areas for intervention targeting agricultural and logging workers, entomological surveillance, and macaque surveillance. Before the development of a priority zone map for intervention targeting agricultural and forest workers, the land cover of the workplace of agricultural and logging workers was estimated by overlaying the covariate layers of cropland, oil palm, and historical tree loss. For each pixel grid, the highest value of either of the overlaid value was selected to represent the value of the output map. A priority zone map highlighting important areas for intervention targeting agricultural and logging workers is important as this group of populations is considered at-risk and regularly exposed to potentially infective mosquitoes (Grigg et al., 2017; Chin et al., 2021). It was noted that 92% of tree cover loss in the year 2010–2019 was driven by deforestation (Global Forest Watch, 2021). Hence, it is important to consider the high likelihood of logging workers presence in areas where tree loss occurred. The relative occurrence probability maps of the *Anopheles* Leucophyrus group mosquito, *M. fascicularis*, and *M. nemestrina* were included in the development of priority zone maps for entomological and macaque surveillance. Threshold values indicating relative priority scores were set based on the quantile-based classification of each covariate and predicted risk map. We assigned the values in the first and second quarters a score of 0, values in the third quarter a score of 1, and values in the fourth quarter a score of 2. The score assignment of each covariate and risk map was described in Supplementary Table 5. For each objective, the relative priority score of covariates and predicted risk map were summed to produce scores ranging between 1 (lowest priority) to 5 (highest priority).

## 3. Results

### 3.1. Model development and evaluation

**Figure 3:**
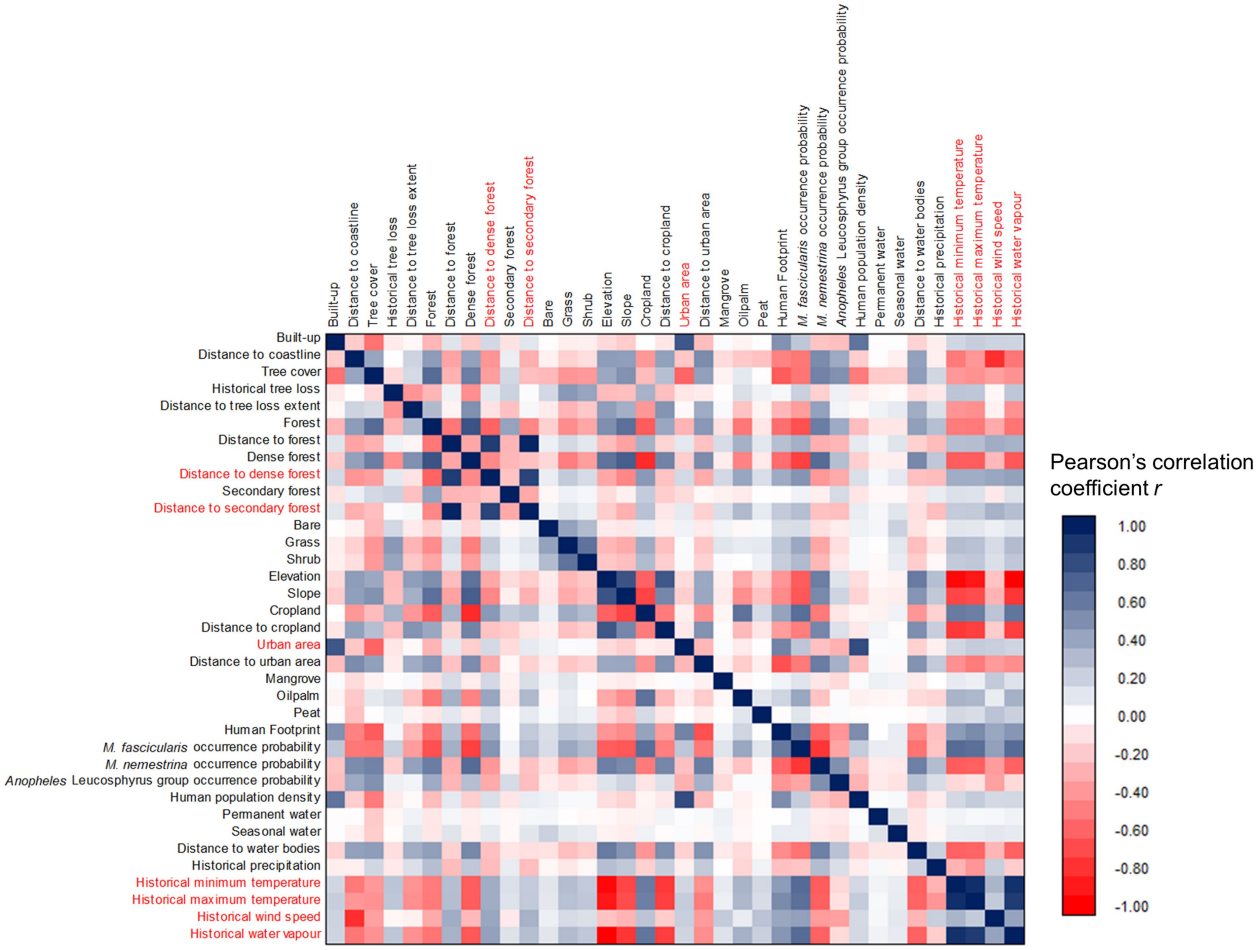
Correlation matrix of all spatial covariates. Covariates highlighted in red indicated high collinearity (*r* ≤ −0.8 or ≥ 0.8) with at least one of other covariates were removed from the modeling dataset.

Multicollinearity assessment *via* a pairwise correlation matrix revealed strong correlations between several covariates (Figure 3). Seven covariates with strong correlation relationships were removed while retaining relevant covariates in the modeling dataset. For instance, elevation has strong a negative correlation with three spatial climate covariates (historical minimum temperature with *r* = −0.93, historical maximum temperature with *r* = −0.88, and historical water vapor with *r* = −0.97). Thus, elevation is deemed more suitable to be maintained to represent these climate covariates. Besides, dense forest and secondary forest covariates were removed to ensure that the dataset achieved an overall VIF <10. Twenty-seven covariates were maintained for subsequent analysis after multicollinearity assessment. Before modeling, the human population density was excluded for inclusion as a sampling bias layer, leaving a balance of 26 covariates as predictors in starting model.

Backward stepwise elimination was conducted by initial MaxEnt modeling using 26 spatial covariates. Subsequently, we identified a reduced dataset of 14 covariates which fulfilled the criteria of having a percent contribution of ≥1 (Table 1). MaxEnt modeling using the final covariate dataset depicted high model performance with mean AUC_ROC_ values of 0.835 ± 0.003 and 0.824 ± 0.007 for train and test datasets, respectively, (Table 2). The most important covariates were distance to coastline, forest cover, cropland, *M. fascicularis* occurrence probability, historical tree loss, and historical annual precipitation (Table 1).

**Table 1:** Relative importance of each covariate toward modeling of human knowlesi malaria risk based on MaxEnt model percent contribution.

Covariates	Percent contribution
Distance to coastline	22.643 ± 1.667
Forest cover	17.687 ± 2.555
Cropland	11.120 ± 2.600
*M. fascicularis* occurrence probability	9.634 ± 0.818
Historical tree loss	6.732 ± 1.219
Historical annual precipitation	5.681 ± 0.712
Oil palm	5.594 ± 1.876
Tree cover	4.493 ± 0.876
Elevation	3.980 ± 1.534
Human footprint	3.319 ± 1.219
Built-up	2.910 ± 0.298
Distance to cropland	2.506 ± 0.899
Distance to forest	2.337 ± 0.891
*M. nemestrina* occurrence probability	1.366 ± 0.306

**Table 2:** Performance comparison across MaxEnt, XGBoost, and ensemble models.

Model	MaxEnt		XGBoost		Ensemble	
Dataset	Train	Test	Train	Test	Train	Test
AUC_ROC_	0.833 ± 0.003	0.821 ± 0.009	**0.933 ± 0.002**	**0.854 ± 0.007**	0.904 ± 0.002	0.845 ± 0.008
Sensitivity	0.622 ± 0.006	0.606 ± 0.026	**0.916 ± 0.004**	**0.742 ± 0.18**	0.781 ± 0.005	0.684 ± 0.020
Specificity	**0.874 ± 0.003**	**0.874 ± 0.003**	0.816 ± 0.003	0.816 ± 0.003	0.848 ± 0.003	0.848 ± 0.003
F1-score	0.479 ± 0.007	**0.312 ± 0.008**	**0.548 ± 0.005**	0.293 ± 0.005	0.527 ± 0.005	0.308 ± 0.005

Bolded value indicates the best performance per evaluation metric (AUC_ROC_, Sensitivity, Specificity, and F1-score) per train or test dataset across the three modeling methods.

**Figure 4:**
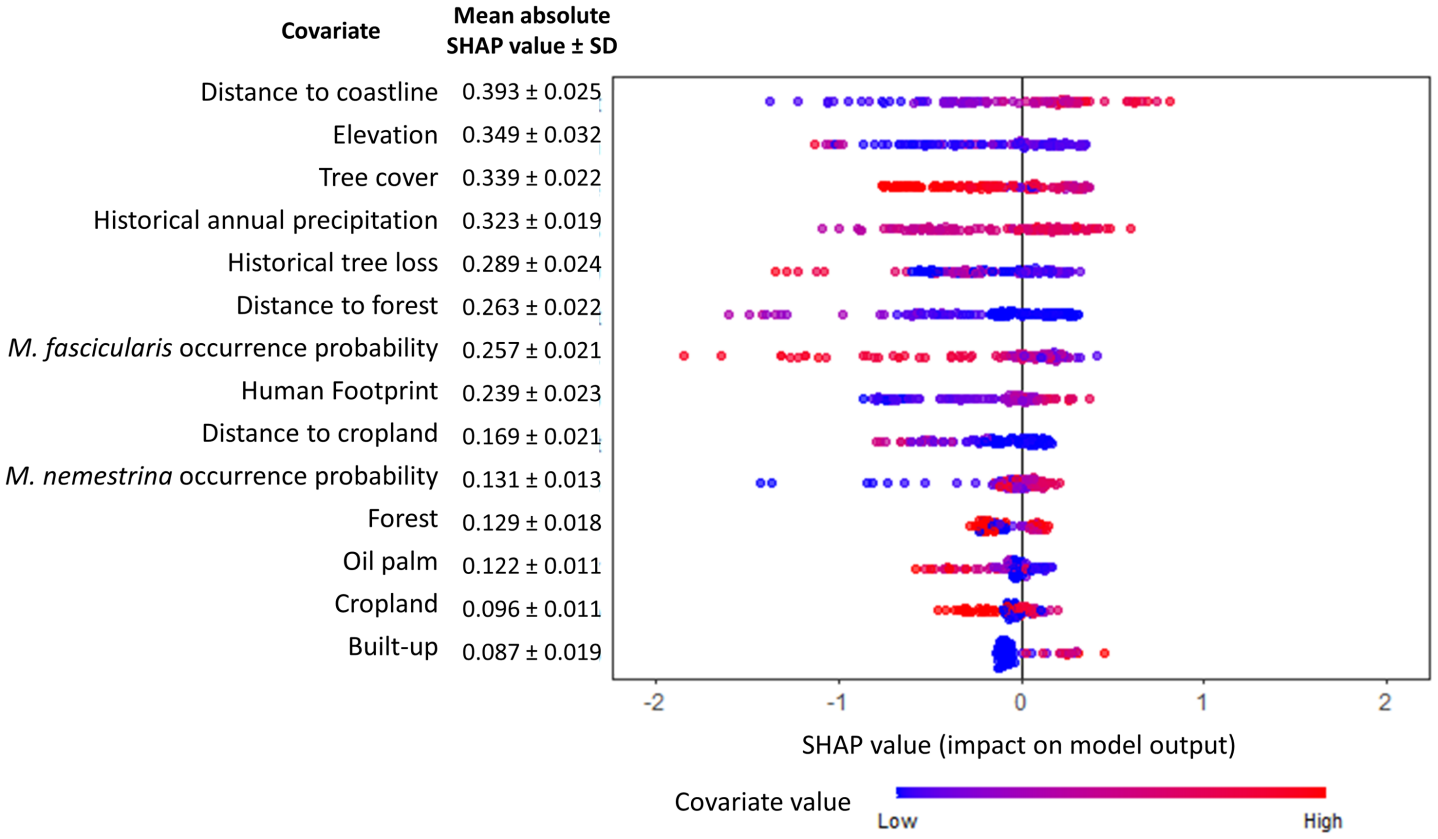
Global SHAP summary plot. The relative importance of each covariate toward human knowlesi malaria risk is indicated and ordered (most important covariate at the top) by the mean absolute SHAP value summarized over 30 model replicates. Warmer dot color indicates higher value of corresponding covariate.

**Figure 5:**
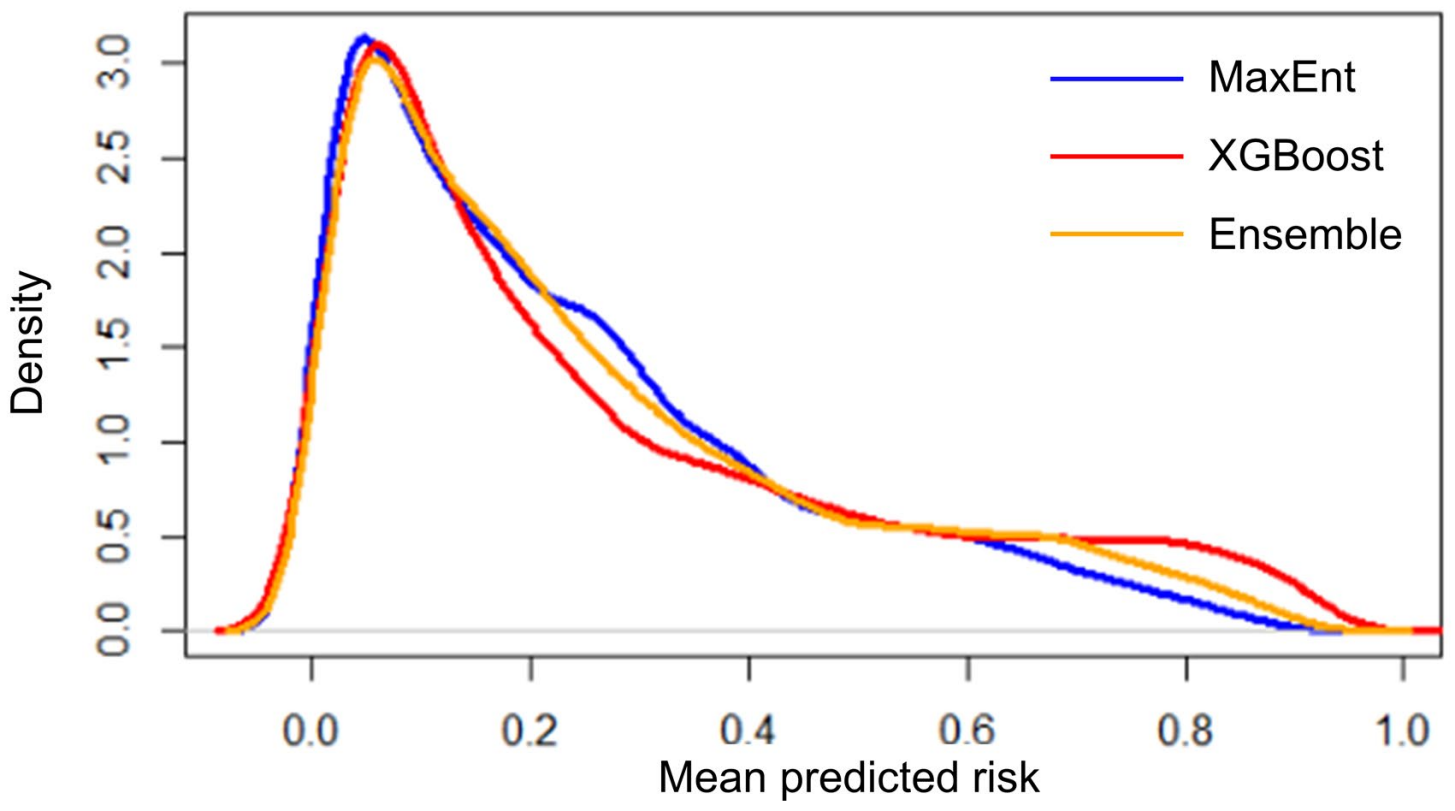
Distribution of mean predicted risk of each model output based on kernel density estimation. Spearman’s correlation test was conducted for MaxEnt-XGBoost (*ρ* = 0.899, value of *p* < 0.001), MaxEnt-ensemble (*ρ* = 0.969, value of *p* < 0.001), and XGBoost-ensemble (*ρ* = 0.977, value of *p* < 0.001).

XGBoost modeling using the final 14 covariates showed high predictive performance with AUC_ROC_ values of 0.933 ± 0.002 and 0.854 ± 0.007 for the train and test datasets, respectively, (Table 2). The key covariates in the model fitting of XGBoost were distance to coastline, elevation, tree cover, historical annual precipitation, historical tree loss, and distance to forest (Figure 4). The output of ensemble model built showed higher AUC_ROC_ than MaxEnt but lower than XGBoost (AUC_ROC_ = 0.904 ± 0.002 for train dataset and AUC_ROC_ = 0.845 ± 0.008 for test dataset). Despite XGBoost having a superior performance as compared to the other models, kernel density estimation showed a relatively similar distribution of predicted risk across models. There were statistically significant high positive correlations for all pairwise comparisons of the models: MaxEnt-XGBoost (*ρ* = 0.899, value of *p* < 0.001), MaxEnt-ensemble (*ρ* = 0.969, value of *p* < 0.001), and XGBoost-ensemble (*ρ* = 0.977, value of *p* < 0.001) (Figure 5).

### 3.2. Environmental suitability for the occurrence of human knowlesi malaria

**Figure 6:**
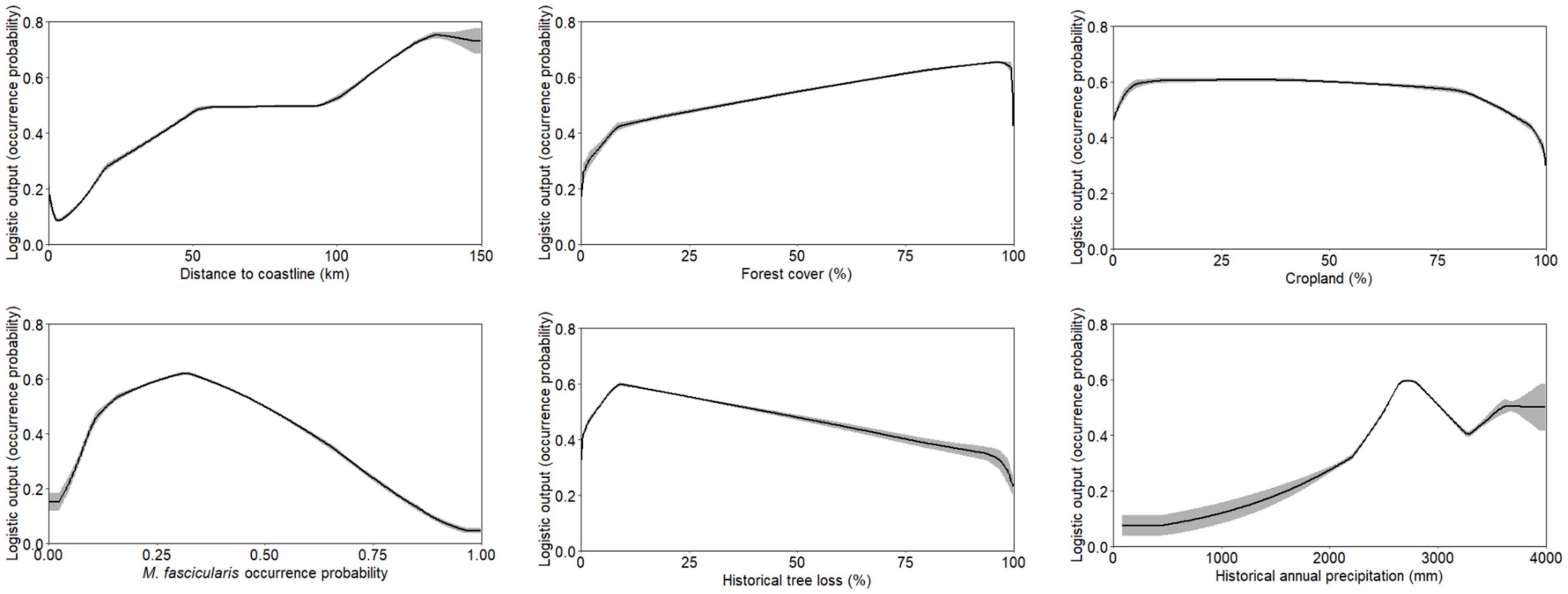
Response curve outputs from MaxEnt model demonstrating the range of suitability for human knowlesi malaria occurrence based on only key covariates with highest model percent contribution as described in Table 1. Grey band indicates standard deviation of the model output.

**Figure 7:**
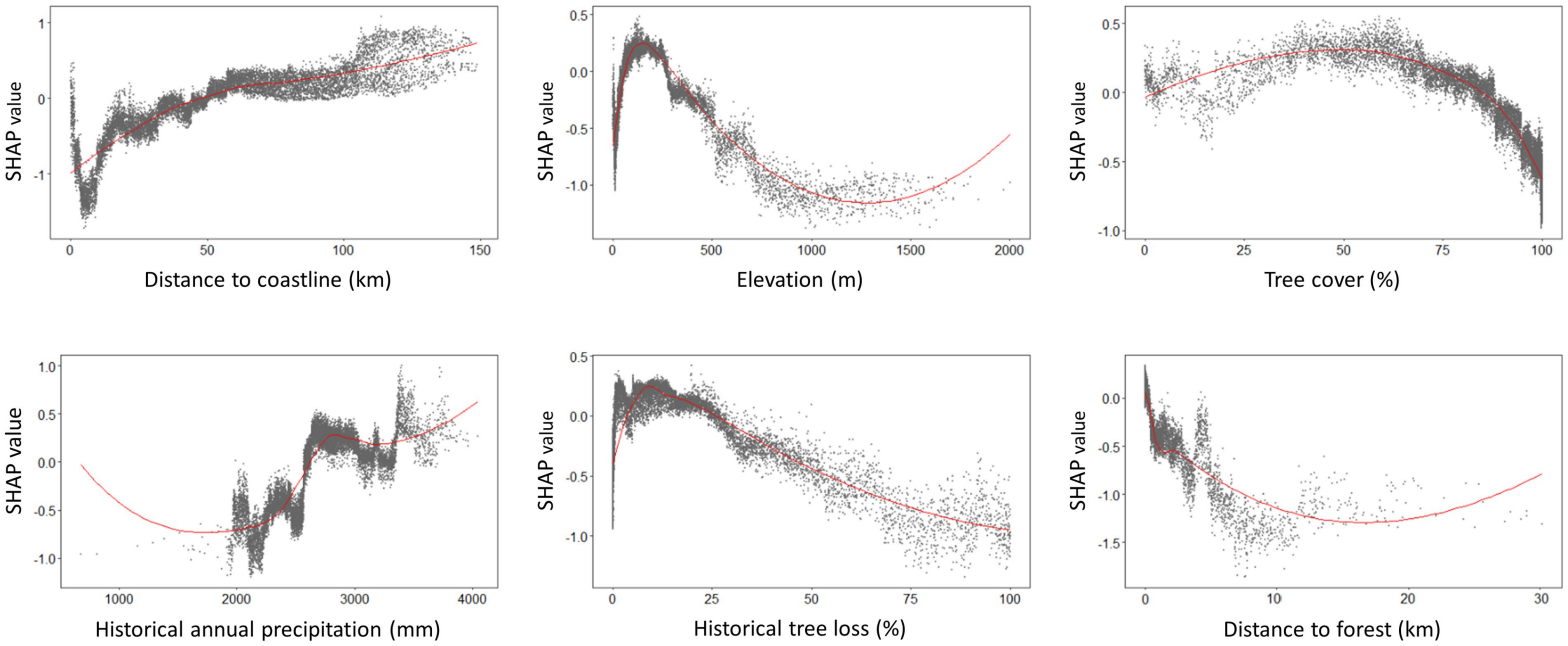
SHAP dependence plots generated from XGBoost model demonstrating the environmental suitability range for human knowlesi malaria occurrence based on only key covariates with highest mean absolute SHAP value as reported in Figure 4. Positive SHAP value indicates higher risk of knowlesi malaria infection whereas negative SHAP value indicates lower risk of knowlesi malaria infection. The plots were smoothed using LOESS (locally estimated scatterplot smoothing) curve in red.

Suitable range of each important environmental factor for the occurrence of human knowlesi malaria was identified based on the response curve of MaxEnt model and the partial dependence plot of XGBoost model (Figures 6, 7). Both models indicated that there was a higher risk of human knowlesi infection at inland areas distant from the coastline (>50 km distance in XGBoost or > 70 km distance in MaxEnt), experienced low intensity of tree loss (3–20% in XGBoost or 3–40% in MaxEnt), and with high annual precipitation (>2,500 mm in MaxEnt or > 2,640 mm in XGBoost). XGBoost demonstrated that there was a higher risk of human knowlesi malaria infection at lower elevation regions of 75–345 m above mean sea level, a wide range of tree cover (<82%), and near to forest landscape (<200 m). In association with various forest-related covariates, MaxEnt showed that the risk of knowlesi malaria increased at >32% forest cover.

As various forest-related covariates (forest cover, tree cover, historical tree loss, and distance to forest) were found to have significant influences on either of the two models, it was of interest to identify the type of forest where knowlesi malaria transmission is high. Thus, an alternative dataset was prepared by replacing the tree cover and forest cover with dense forest cover and secondary forest cover. An XGBoost analysis involving this dataset showed that knowlesi malaria cases have a higher probability to occur in areas with high secondary forest cover (>13%) and with low dense forest cover (<18%) (Supplementary Figure 4).

Besides, the knowlesi malaria environmental suitability range was found to be influenced by other spatial attributes such as *M. fascicularis* occurrence probability, and cropland in MaxEnt (Figure 6). This signifies that the occurrence of human knowlesi malaria has a specific ecological niche with multi-dimensional environmental factors playing roles in the disease transmission cycle.

### 3.3. Distribution of human knowlesi malaria in Peninsular Malaysia

**Figure 8:**
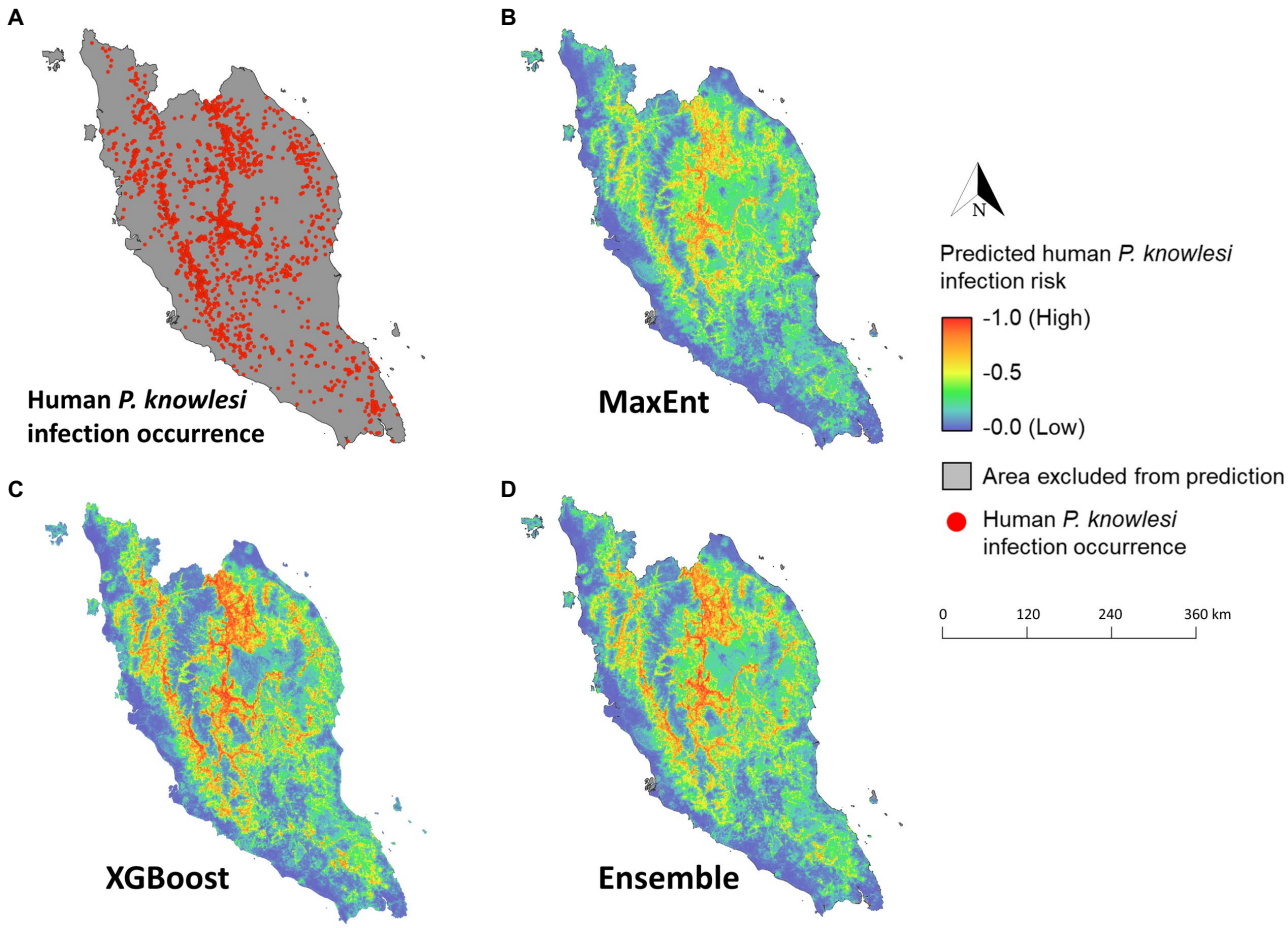
Maps of predicted human knowlesi malaria risk in Peninsular Malaysia. **(A)** Map of geolocated human knowlesi malaria occurrence throughout Peninsular Malaysia from years 2011–2019. Risk maps generated by MaxEnt **(B)**, XGBoost **(C)**, and ensemble models **(D)**. Warmer color indicates higher predicted risk of knowlesi malaria.

**Figure 9:**
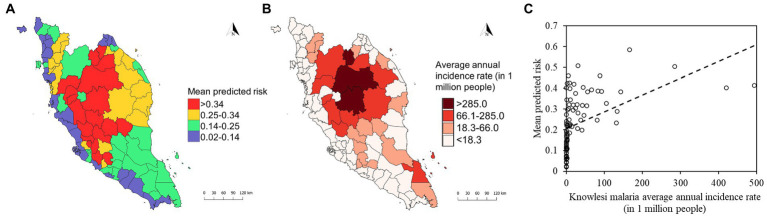
**(A)** District-level mean predicted risk of human knowlesi malaria based on XGBoost output. **(B)** Average annual incidence rate of knowlesi malaria from 2011 to 2019 by district. Color gradations for maps in **(A,B)** were determined using Jenks natural breaks. **(C)** Correlation plot shows statistically significant positive correlation (Spearman’s correlation coefficient *ρ* = 0.76, value of *p* < 0.001) between mean predicted risk and knowlesi malaria average annual incidence rate (in 1 million people).

The mean model outputs were used to generate predicted human *P. knowlesi* infection risk maps of 1×1 km^2^ pixel spatial resolution (Figure 8). All models generated similar predicted spatial patterns across Peninsular Malaysia. Risk map generated by XGBoost was used as the final map output due to its higher performance compared to other models (Table 2). Based on the risk map, the models predicted that the ecological factors in the central-northern region of Peninsular Malaysia and the lower elevation areas along Titiwangsa mountain range are highly suitable for knowlesi malaria transmission. The mean predicted risk value was extracted for each district in Peninsular Malaysia. The district-level mean predicted risk is presented alongside the average annual human knowlesi malaria incidence rate in year 2011–2019 (Figures 9A,B). There is a significant positive correlation between mean predicted risk and disease incidence rate (in 1 million people) (Spearman’s correlation coefficient *ρ* = 0.76, value of *p* < 0.001; Figure 9C).

### 3.4. Intervention and surveillance priority zone maps

**Figure 10:**
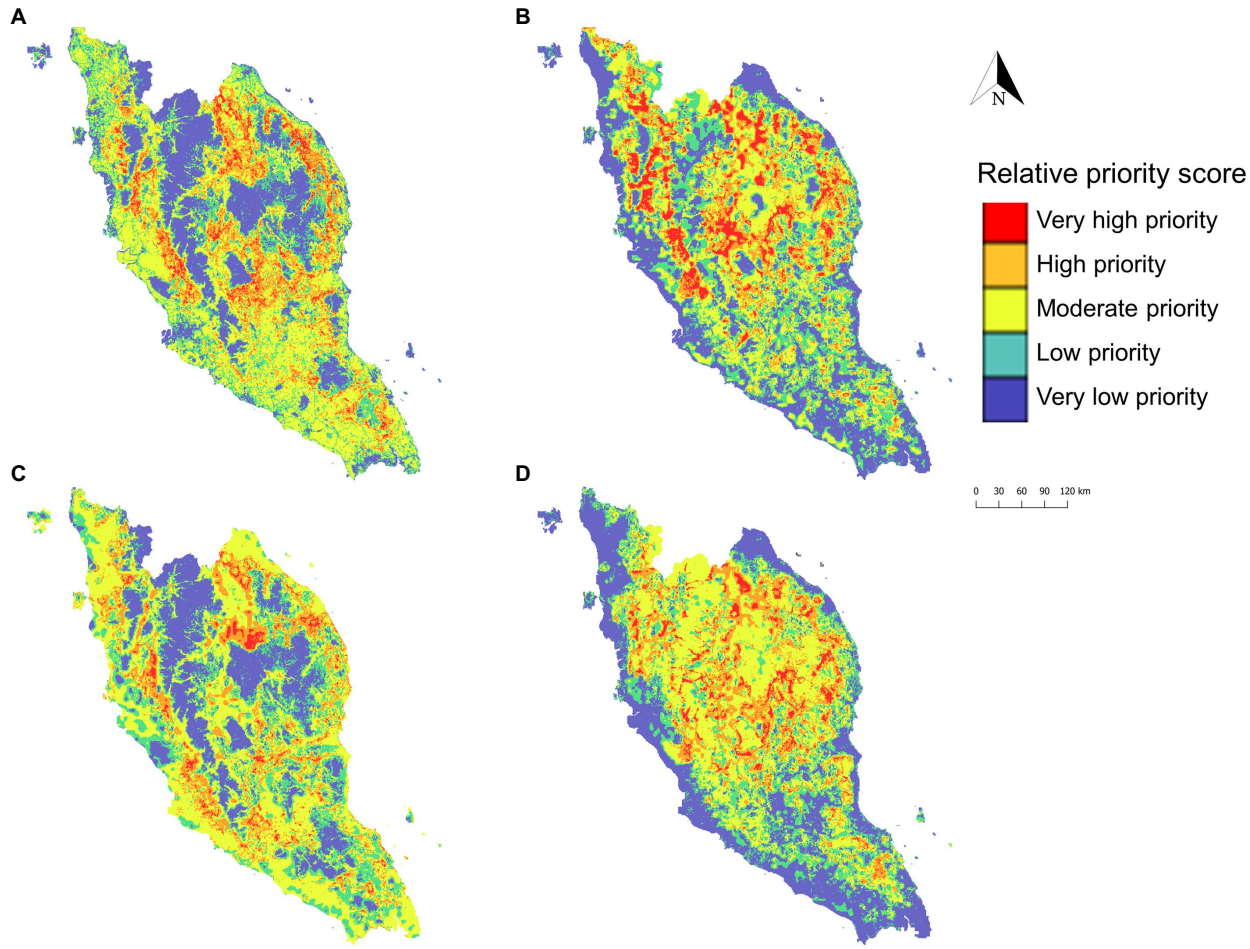
Priority zone maps developed to identify priority areas for intervention targeting agricultural and logging workers **(A)**, *Anopheles* Leucosphyrus group vector surveillance **(B)**, *M. fascicularis* surveillance **(C)**, and *M. nemestrina* surveillance **(D)**. The priority zone was determined using the sum of relative priority score of respective covariate spatial layer and XGBoost predicted risk of human knowlesi malaria.

The predicted risk map produced using XGBoost was subsequently selected for developing the intervention and surveillance priority zone maps (Figure 10). In coherence with the predicted risk map, most of the high-priority areas are situated in the central northern region of Peninsular Malaysia. For surveillance targeting agricultural and logging workers, the high-priority zones are mostly located in suburban areas in the central-northern Peninsular Malaysia region as well as near hills in the southern state of Johor (Figure 10A). *Anopheles* Leucosphyrus group mosquito priority zone maps indicated that key areas for enhanced surveillance are mostly located in the interior (Figure 10B). *M. fascicularis* surveillance priority zones are mainly situated in the peri-domestic areas as compared to *M. nemestrina* surveillance priority zones, which are mainly found in the interior part of Peninsular Malaysia.

## 4. Discussion

This study incorporated diverse environmental data sources as well as the national knowlesi malaria case data to predict spatial knowlesi malaria transmission risk using machine learning approaches. Higher performance was observed in XGBoost as compared to other modeling approaches. XGBoost can generate high-resolution maps showing the risk of knowlesi malaria transmission to humans from known reservoirs, specifically *M. nemestrina* and *M. fascicularis*. One of the primary benefits of this map is that it allows for the identification of high-risk areas down to the village level. These high-risk areas can be prioritized for intervention or strengthening of existing surveillance systems.

In understanding the spatial heterogeneities of human knowlesi malaria occurrence, it is important to identify diverse environmental factors with optimal ranges that drive the transmission. For instance, forest cover was recognized as a key predictor in the MaxEnt model training, which reflects the role of forest environments as the habitats of macaque reservoirs and *Anopheles* mosquito vectors. Likewise, the XGBoost model showed that knowlesi malaria risk is higher in and near to the forest, which has also been observed in previous studies (Tan et al., 2008). A study in Sarawak found that the *P. knowlesi* vector *An. latens* had the highest sporozoite and oocyst rates in the forest as compared to farms (Tan et al., 2008). The association of increased knowlesi malaria occurrence with both forest and forest loss provides further support for the hypothesis that transmission occurs in forested areas undergoing substantial change (Fornace et al., 2016). Deforestation has been considered the main driver in the transmission of knowlesi malaria. As shown in this study, further classification of forest into dense forest and secondary forest revealed that the risk of knowlesi malaria is higher in areas mainly covered with secondary forest. An entomological study in Sabah found that the abundance of the local primary vector of knowlesi malaria, *An. balabacensis* is higher in the logged forest as compared to the primary forest (Brant et al., 2016). Another study revealed that higher percentage of infectious bites were likely to occur at households at forest edges (Fornace et al., 2019). This is related to the anthropogenic-induced conversion of forests into other land use such as cropland and settlements, which would affect macaque movements (Stark et al., 2019). For instance, the movement of macaques from forests to plantations and human settlements for food foraging would increase the contact between humans and macaques as well as the probability of zoonotic transmission of *P. knowlesi* can occur in the presence of efficient vectors (Imai et al., 2014).

In general, the predicted high-risk areas of knowlesi malaria are concentrated in lower elevation areas along the Titiwangsa mountain range and the central-northern region of Peninsular Malaysia. Other studies also indicated that geographical elevation was negatively associated with knowlesi malaria exposure (Fornace et al., 2016, 2019). This is because both the macaque hosts and vectors are more frequently found at lower elevation (Fooden, 1995). The risk of knowlesi malaria occurrence increased relative to distance from the coastline. This is apparent as forested areas where high transmission occurs are mainly situated inland. Greater urbanization nearer to the coastline has disrupted *Anopheles* mosquitoes’ habitat and abundance, thus, transmission intensity in these areas is likely low (Ferraguti et al., 2016).

Both MaxEnt and XGBoost models explain that knowlesi malaria tends to occur in areas with high historical annual precipitation. Consistent rainfall with partial contribution from land-use changes would create favorable breeding sites for *Anopheles* mosquitoes and support larval development (Oo et al., 2002; Ahmad et al., 2018). In Sabah, an increase in knowlesi malaria cases was observed after 2 to 4 months of increased rainfall (William et al., 2014). Also, an increase in knowlesi malaria incidence 3 months after higher rainfall and higher humidity was found *via* univariate analyses in another study, but these associations were not statistically significant in multivariate analysis (Cooper et al., 2020). In Thailand, climate factors such as rainfall, temperature, and relative humidity were found to be associated with malaria incidence (Kotepui and Kotepui, 2018). Extreme rainfall may be unfavorable to malaria transmission as it would lead to a wash-out effect that disrupts vector breeding sites and causes larvae mortality (Thomson et al., 2005; Tompkins and Ermert, 2013). The utilization of time-series modeling would be able to help in explaining the non-linear relationship between rainfall and malaria transmission in detail. Also, there was a transient drop of number of knowlesi malaria cases throughout Malaysia in year 2015 and 2016, which was thought to be impacted by changing weather pattern and El Niño phenomenon (Cooper et al., 2020; Phang et al., 2020; Ooi et al., 2021). Nevertheless, other factors such as landscape factors such land-use change and deforestation play important roles in transmission patterns, which makes it difficult to fully understand the impact of climate change on knowlesi malaria transmission. More research is needed to fully understand the complex relationship between climate change and *P. knowlesi* transmission.

The influence of *Anopheles* Leucosphyrus group mosquito occurrence was found to be less important in our models. This covariate was initially modeled using the scattered data collected before 2013 which may not present the reliable spatial distributions in the study region and resulted in its weak association with disease occurrence (Moyes et al., 2016). Breeding behavior, abundance, and distribution of certain mosquito species may change drastically over time due to landscape shifts, deforestation, and human encroachment (Burkett-Cadena and Vittor, 2018). At present, only *Anopheles* Leucosphyrus group mosquitoes are recognized as the vector of *P. knowlesi* in Peninsular Malaysia, but recent studies conducted in Sarawak have added *An. donaldi* from the Barbirostris group as well as *An. collessi* and *An. roperi* from the Umbrossus group into the list of potential vectors (Ang et al., 2020, 2021). It may be possible that there are efficient vectors other than the Leucosphyrus group mosquitoes in Peninsular Malaysia. It is necessary to implement continuous entomological surveillance for updating entomological data to monitor changes in *Anopheles* mosquito biology, to identify potentially new vectors, as well as to investigate the possible influence on receptivity across multiple localities in Malaysia. In addition, new tools are essential to enable efficient and cost-effective entomological fieldwork. For instance, the predictive risk map developed in this study has the potential to guide entomologists in identifying suitable surveillance locations. To complement the efficiency of vector sampling in the field, the use of commercialized mosquito traps as a safer alternative to human landing catch and the application of multiplex polymerase chain reaction assay for the accurate identification of certain *Anopheles* mosquito species should be considered (Jeyaprakasam et al., 2021b; Pramasivan et al., 2022).

The utility of MaxEnt has been well documented in various epidemiology-related ecological studies for its high performance in species distribution range prediction. However, this showed that XGBoost performed better than MaxEnt. Nevertheless, this may not indicate that XGBoost always offers superior performance compared to MaxEnt. This is because each model has different strengths and weaknesses with different outcomes. Therefore, an ensemble of multiple models is recommended to integrate the attributes of each involved model in a complementary manner. This approach is generally applied to address issues such as incremental learning, imbalanced data, error correction, and confidence estimation, and it usually generates improved results (Polikar, 2012). Some studies highlighted that combining relatively high-performing base models with low correlation or high diversity can generate ensemble models with higher performance (Pan et al., 2019; Yu et al., 2022). Nonetheless, our study demonstrated that the use of a single best-performing base model of XGBoost was adequate because the outputs from both base models, MaxEnt and XGBoost, were highly correlated with a lack of novel information to improve ensemble model performance.

The approach applied in this study demonstrated the importance of integrating empirical data from multiple agencies and developed a guide for future collaborative-based programs. From the zoonotic malaria control perspective, it is important to address the interdependence between humans, animals, and their environmental variations. The involvement of macaques as the natural hosts of *P. knowlesi* complicates the elimination and subsequent eradication of malaria and requires intervention strategies designed to specifically address zoonotic pathways, which is different from the strategy for tackling human malaria (Vythilingam et al., 2018; Mohammad et al., 2022). Thus, a unifying approach converging transdisciplinary and multisectoral efforts is essential to combat the transmission of *P. knowlesi*, as advocated in the “One Health” concept. These efforts include sharing and co-assessment of intervention and data from epidemiologists, clinicians, zoologists, and entomologists, development of novel tools and platforms that can be adapted in different settings, as well as converging diagnostics for human, vector, and macaque reservoirs.

The development of intervention and surveillance priority zone map highlighted how the risk map can be further utilized to identify priority areas for concentrated efforts. For instance, the localities of the population at risk can be identified and effective interventions can be adapted to target populations. In this case, personal-level protective equipment such as insecticide-treated outdoor clothing, topical repellent, chemoprophylaxis, and spatial repellent shall be distributed more frequently to agricultural and logging workers, military personnel, as well as people living in high-risk areas (Vythilingam et al., 2021; Mohammad et al., 2022). Regular screening as well as awareness programs shall be conducted for communities in these areas. Specifically, in high-risk areas with a lack of accessible routes, the development and distribution of highly sensitive, mobile, and affordable tools such as novel rapid diagnostic test kits will enhance public health outreach (Tan et al., 2022).

Several potential strategies have been highlighted in relation to vector and wildlife controls. At present, indoor residual spraying and insecticide-treated net have been practiced as the core vector interventions in Malaysia (Ministry of Health Malaysia, 2022). However, the effectiveness of certain indoor-based interventions may be limited by the outdoor biting behaviors of the *P. knowlesi* vectors (Grigg et al., 2017; Vythilingam et al., 2021). Recent studies showed that outdoor-based applications such as outdoor residual sprays are effective against primary *P. knowlesi* vectors in Malaysian Borneo (Rohani et al., 2020, 2021). The distribution of vaccines or drug-treated oral baits for macaques has been proposed in wildlife-based intervention, and it is less invasive than macaque population culling, which is being debated for ethical reasons and uncertain implications (Cuenca et al., 2021). This similar method has been found promising in controlling other zoonoses such as Lyme disease (Dolan et al., 2017) and rabies (Rosatte et al., 2009; Maki et al., 2017). Nonetheless, there are currently no suitable vaccine or drug candidates that could be adapted for similar use in knowlesi malaria wildlife control programs. The use of oral baits will necessitate further research, and as suitable oral baits are developed in the future, they can be distributed to macaque populations in knowlesi malaria high-risk areas.

Surveillance, monitoring, and intervention are important aspects of zoonotic disease management and control because they serve as a guideline for detecting high-risk areas early in an outbreak and deciding how to allocate resources and manpower during disease outbreaks. The generated risk map had a high level of agreement with the actual data. Therefore, zoonotic disease management and control efforts should be targeted at the areas showing high probability of human knowlesi malaria occurrence. Furthermore, we propose that covariates with a high contribution be considered in field monitoring. We can identify the relative impact of environmental factors on knowlesi malaria occurrence by analyzing the partial dependence plots of each model. This data is required for epidemiologists, public health officials, and policymakers to effectively monitor and control knowlesi malaria.

There are several limitations to address concerning this study. Firstly, the ecological niche modeling approach in this study did not specifically consider the spatial variability of *P. knowlesi* infections in macaques and mosquitoes. To develop a surveillance system of macaques and vectors at priority zones will provide such information to enhance the accuracy of risk maps. Secondly, moderate F1-scores, which is caused by imbalanced data and random selection of background data near to reported cases, produced more false positive predictions. Elevated false positive rates may place additional demands on resources for monitoring and managing disease, however, this can be systematically reduced by alternative methods of identifying priority zones for targeted interventions. In addition, advanced deep learning algorithms can be considered to enhance model performance in the future.

## 5. Conclusion

Machine learning-based ecological niche modeling approaches such as MaxEnt and XGBoost are extremely useful in capturing diverse ecological signals relevant to spatial distributions of vector-borne diseases. The predictive risk maps produced in the present study can be used to identify high-risk areas of knowlesi malaria transmission and provide more precise information for decision-making of vector or reservoir surveillance and disease control, particularly when prevention resources are limited.

## Data availability statement

The data analyzed in this study is subject to the following licenses/restrictions: The data of this study are available from the Ministry of Health Malaysia. Restrictions apply to the availability of these data. Data are available with the permission of the Ministry of Health Malaysia. The data generated in this study is available from the corresponding author on reasonable request. Requests to access these datasets should be directed to chtingwu@tmu.edu.tw.

## Ethics statement

The studies involving human participants were reviewed and approved by registered with the National Medical Research Register (NMRR-16-2109-32928), and ethical approval was obtained from the Malaysian Research Ethical Committee (MREC) [reference no. KKM/NIHSEC/P16-1782 (11)]. For all case data, information that identifies the patient was anonymized. Written informed consent from the participants’ legal guardian/next of kin was not required to participate in this study in accordance with the national legislation and the institutional requirements.

## Author contributions

WP, T-WC, YL, and MF conceptualized and designed the study. MH, JJ, and RM were involved in data collection and provided the dataset for analysis. WP and T-WC conducted the data analysis. WP wrote the manuscript. All authors critically reviewed, revised, and approved the final manuscript.

## Funding

This study was supported by the Ministry of Higher Education, Malaysia Long Term Research Grant Scheme (LRGS/1/2018/UM/01/1/1) and the Ministry of Science and Technology, Taiwan (MOST110-2621-M-038-001-MY2).

## Conflict of interest

The authors declare that the research was conducted in the absence of any commercial or financial relationships that could be construed as a potential conflict of interest.

## Publisher’s note

All claims expressed in this article are solely those of the authors and do not necessarily represent those of their affiliated organizations, or those of the publisher, the editors and the reviewers. Any product that may be evaluated in this article, or claim that may be made by its manufacturer, is not guaranteed or endorsed by the publisher.
