# Corrigendum: Predicting *Plasmodium knowlesi* transmission risk across Peninsular Malaysia using machine learning-based ecological niche modeling approaches

**DOI:** 10.3389/fmicb.2023.1178864

**Published:** 2023-03-16

**Authors:** Wei Kit Phang, Mohd Hafizi bin Abdul Hamid, Jenarun Jelip, Rose Nani binti Mudin, Ting-Wu Chuang, Yee Ling Lau, Mun Yik Fong

**Affiliations:** ^1^Department of Parasitology, Faculty of Medicine, Universiti Malaya, Kuala Lumpur, Malaysia; ^2^Disease Control Division, Ministry of Health Malaysia, Putrajaya, Malaysia; ^3^Sabah State Health Department, Ministry of Health Malaysia, Kota Kinabalu, Sabah, Malaysia; ^4^Department of Molecular Parasitology and Tropical Diseases, School of Medicine, College of Medicine, Taipei Medical University, Taipei, Taiwan

**Keywords:** *Plasmodium knowlesi*, Peninsular Malaysia, ecological niche modeling, XGBoost, ensemble modeling, maximum entropy

In the published article, there was an error in the **Funding** statement. “This study was supported by the Ministry of Higher Education, Malaysia Long Term Research Grant Scheme (LRGS/1/2018/UM/01/1/1) and the Ministry of Science and Technology, Taiwan (MOST108-2638-H-002-002-MY2).” The correct Funding statement appears below.

