# Supplementary material for: Predicting Plasmodium knowlesi transmission risk across Peninsular Malaysia using machine learning-based ecological niche modeling approaches
Source: Front Microbiol. 2023 Feb 16;14:1126418. doi: 10.3389/fmicb.2023.1126418 (PMC9977793; doi:10.3389/fmicb.2023.1126418)
Supplement: Supplementary file 1 [file Data_Sheet_1.docx]

Supplementary Material

Predicting *Plasmodium knowlesi* transmission risk across Peninsular Malaysia using machine learning-based ecological niche modeling approaches

**Wei Kit Phang^1^, Mohd Hafizi bin Abdul Hamid^2^, Jenarun Jelip^2^, Rose Nani binti Mudin^3^, Ting-Wu Chuang^4*^, Yee Ling Lau^1^, and Mun Yik Fong^1^**

^1^Department of Parasitology, Faculty of Medicine, Universiti Malaya, Kuala Lumpur, Malaysia

^2^Disease Control Division, Ministry of Health Malaysia, Putrajaya, Malaysia

^3^Sabah Health Department, Ministry of Health Malaysia, Kota Kinabalu, Sabah, Malaysia

^4^Department of Molecular Parasitology and Tropical Diseases, School of Medicine, College of Medicine, Taipei Medical University, Taipei, Taiwan

*** Correspondence:** Ting-Wu Chuang: chtingwu@tmu.edu.tw

# Supplementary Data

**Spatial data collation and processing**

ArcGIS Pro version 2.7.2 (Esri, Redlands, CA, USA) and QGIS version 3.6.3 (Open Source Geospatial Foundation, Beaverton, OR, USA) were used to visualize and process spatial data. We acquired spatial environmental data from multiple sources of satellite remote sensing data as described in Supplementary Table 1. Slope layer was computed from the United States Geological Survey Earth Resources Observation and Science (USGS EROS) Shuttle Radar Topography Mission (SRTM) elevation layer and expressed in percent rise. For creation of urban extent layer, polygons were manually created to surround each coordinate point of city with ≥10000 people population according to GeoNames database (GeoNames, 2021). Cells with ≥50% built-up within the polygons were extracted and designated as urban area (with value 1) and urban extent layer was generated subsequently (Balk et al., 2018).

Forest extent raster layer was created based on overlapping extent between JAXA ALOS-PALSAR-2 forest/non-forest extent (year 2017) layer and Copernicus Global Land Service land use tree cover fractional (>60% cover) layer (year 2019). The 60% tree cover threshold was based on International Geosphere Biosphere Programme (IGBP) land cover classification of forest (Loveland and Belward, 1997). Forest extent raster layer was further classified into dense forest and secondary forest. To create the dense forest extent, the overlapping cells between forest extent layer and tree cover (>90% cover) layer were extracted. Cropland extent from United States Geological Survey Global Food Security-Support Analysis Data (USGS GFSAD), oil palm extent, and 2010-2019 tree loss/deforested extent were masked from the resulting layer to produce the dense forest extent layer. Secondary forest extent layer was created by subtracting the forest extent layer with dense forest layer. Supplementary Table 2 depicts the further classification of forest into dense forest and secondary forest. Dense forest is described as primary/intact forest, old growth secondary forest, no human disturbance (eg. deforestation, agricultural land, etc), contiguous area of over 0.5 ha, and ≥90% tree cover (Shimada et al., 2014), whereas secondary forest is fragmented forest, young forest, tree plantation, and ≥60% tree cover.

Proximity maps were created using Proximity tool in QGIS version 3.6.3 (Open Source Geospatial Foundation, Beaverton, OR, USA). A total of seven distance maps were generated from source extent raster layers as described in Supplemental Table 3. In a processed distance map, the value of each pixel indicates the distance (in meter) calculated from the centre of each pixel to the centre of the nearest extent pixel of source raster layer. For example, the value of each pixel in the distance to forest map layer represents the distance between the centre that specific pixel to the centre of nearest pixel with forest presence (1). The coordinate reference systems of all spatial data were projected as World Geodetic System (WGS) 84 / Universal Transverse Mercator (UTM) zone 47N. All covariates were resampled to produce raster layers with 1x1 km^2^ resolution (Supplementary Figures 1, 2, and 3).

# Supplementary Figures and Tables


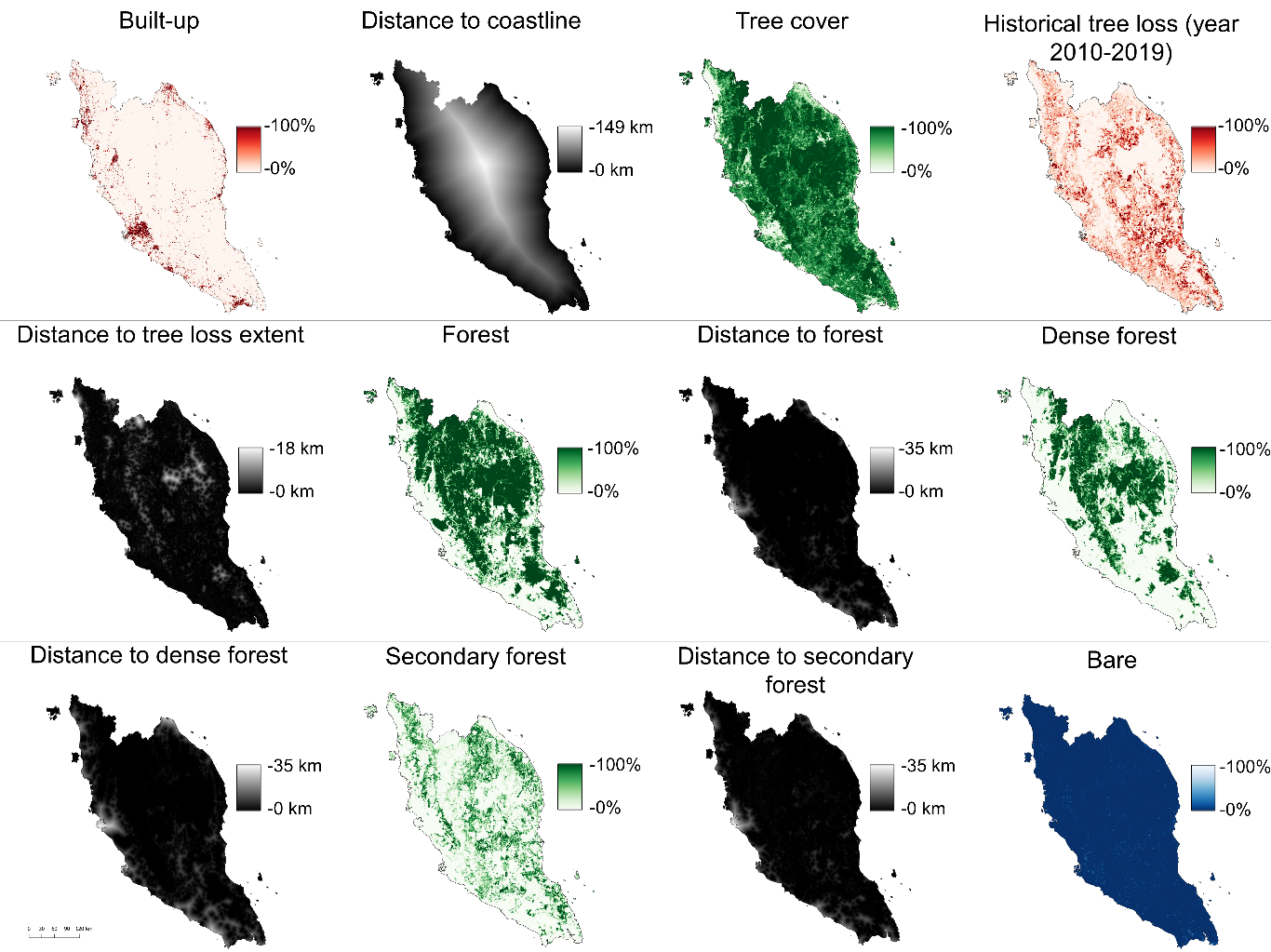


**Supplementary Figure 1.** Processed spatial environmental covariates for modelling procedure.


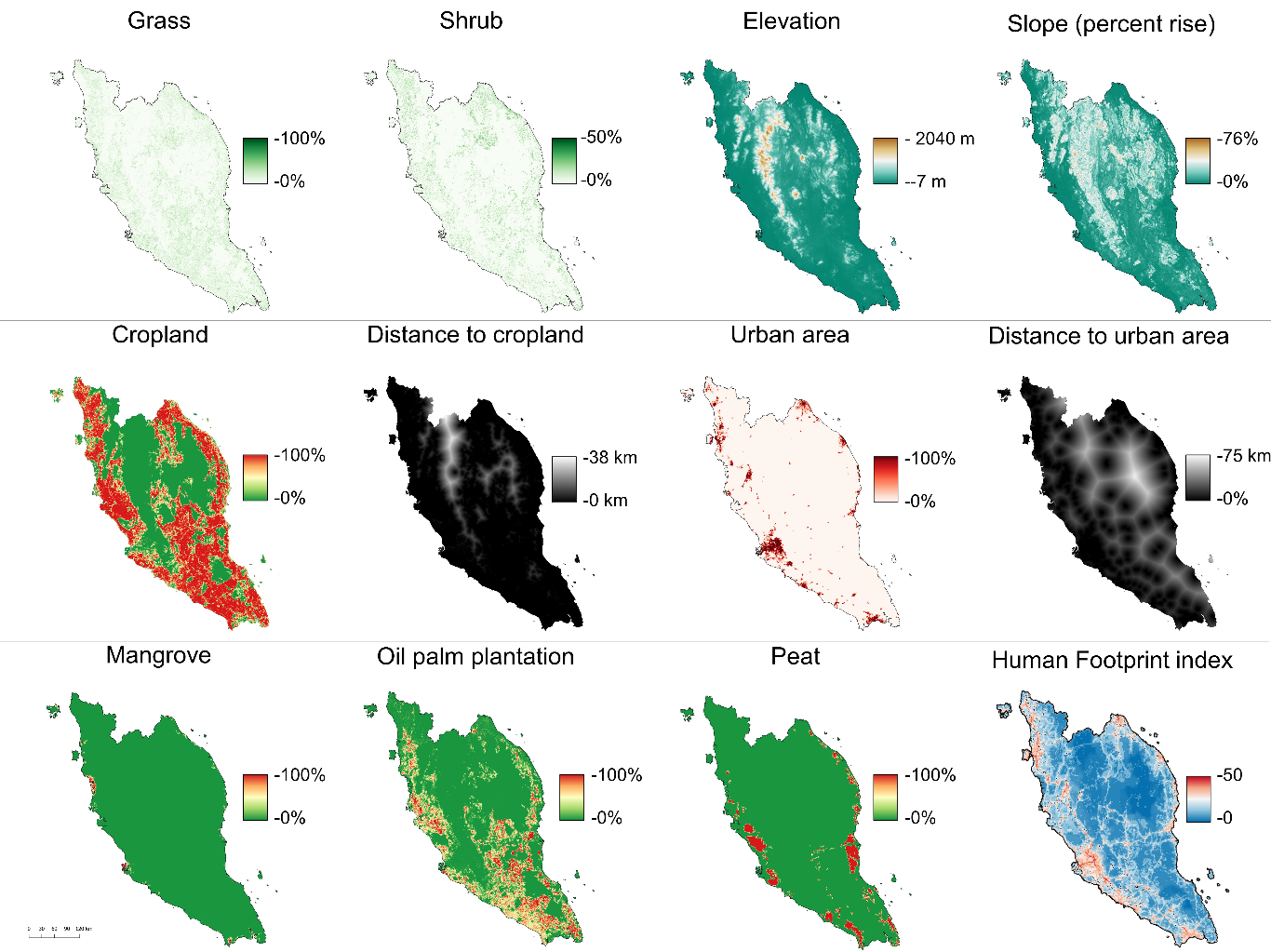


**Supplementary Figure 2.** Processed spatial environmental covariates for modelling procedure.


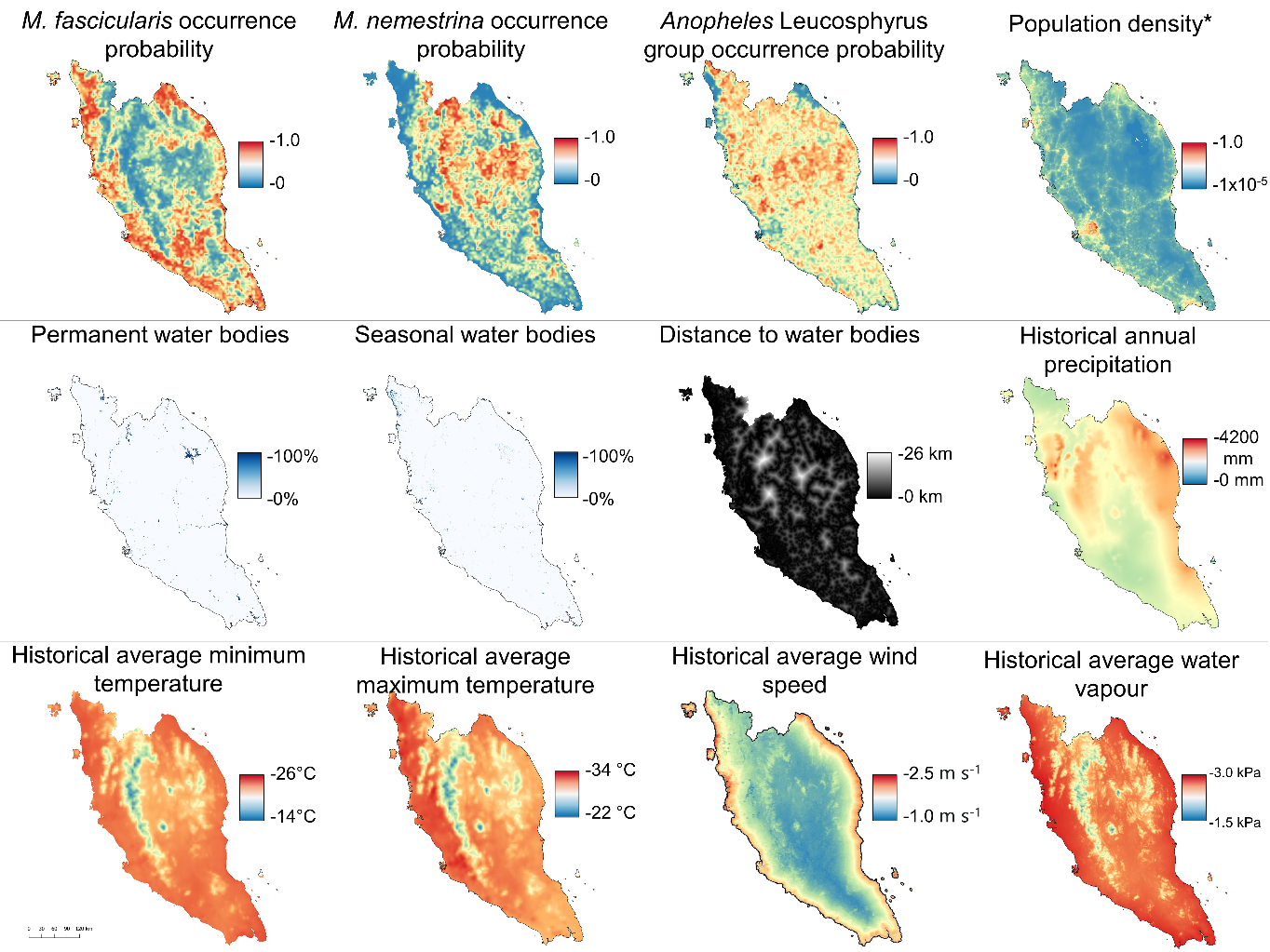


**Supplementary Figure 3.** Processed spatial environmental covariates for modelling procedure. The population density raster layer was log-transformed and rescale to value ranging >0 and ≤1. The population density raster layer was used as bias layer in Maxent modelling and for instance weight information in XGBoost modelling.


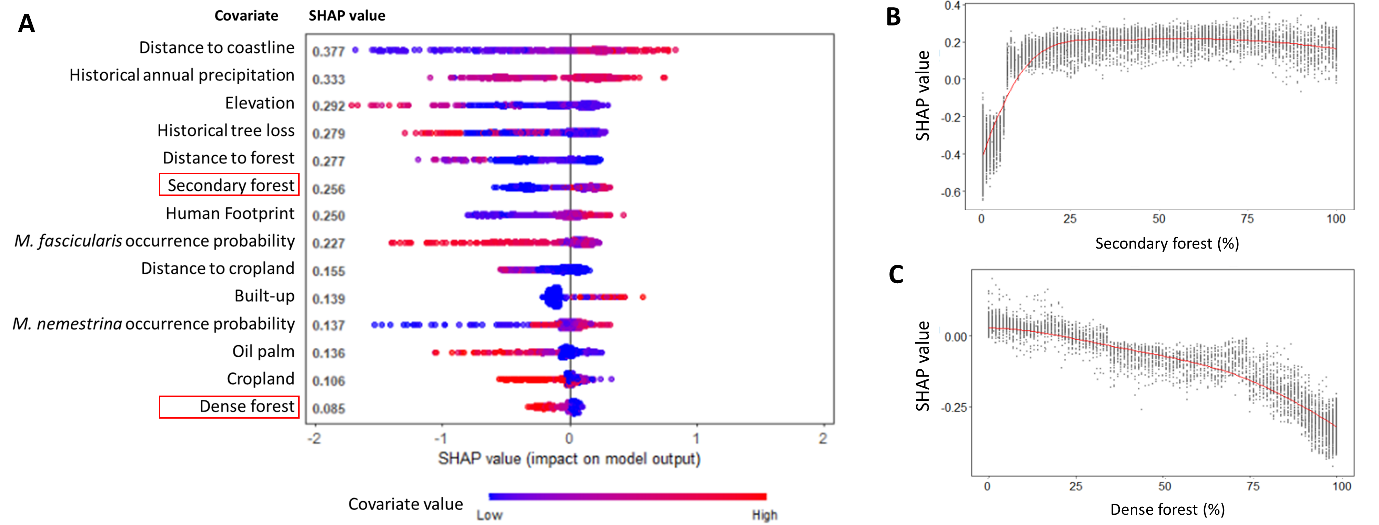


**Supplementary Figure 4.** Summary of XGBoost modelling conducted to investigate the effect of different forest cover classification. Secondary forest and dense forest covariates were included into the model to replace the tree cover and forest in the final dataset. The relative importance of each covariate is indicated and ordered (most important covariate at the top) by the absolute SHAP values (A). The sina plot shows the distribution of covariate contributions to the output of the model. Warmer dot colour indicates higher value of corresponding covariate. Each dot indicates an observation of either case occurrence or background. The AUC_ROC_ of the model training was 0.930. Partial dependence plots indicated that the occurrence probability of human knowlesi malaria is higher at high secondary forest cover (>13%) (B) and at low dense forest cover (<18%) (C).

**Supplemental Table 1.** Spatial environmental covariate data and their sources.

| Covariates | Spatial resolution | Value range | Type | Sources |
| --- | --- | --- | --- | --- |
| Bare (cover fractions) (year 2019) | 100 m | 0 to 100 | Landscape | Copernicus Global Land Service (Buchhorn et al., 2020) |
| Built-up (cover fractions) (year 2019) | 100 m | 0 to 100 | Landscape | Copernicus Global Land Service (Buchhorn et al., 2020) |
| Grass (cover fractions) (year 2019) | 100 m | 0 to 100 | Landscape | Copernicus Global Land Service (Buchhorn et al., 2020) |
| Tree (cover fractions) (year 2019) | 100 m | 0 to 100 | Landscape | Copernicus Global Land Service (Buchhorn et al., 2020) |
| Shrub (cover fractions) (year 2019) | 100 m | 0 to 40 | Landscape | Copernicus Global Land Service (Buchhorn et al., 2020) |
| Permanent water bodies (cover fractions) (year 2019) | 100 m | 0 to 100 | Landscape | Copernicus Global Land Service (Buchhorn et al., 2020) |
| Seasonal water bodies (cover fractions) (year 2019) | 100 m | 0 to 100 | Landscape | Copernicus Global Land Service (Buchhorn et al., 2020) |
| Elevation (m above mean sea level) | 30 m | -4 to 2037 | Landscape | United States Geological Survey Earth Resources Observation and Science (USGS EROS) (Earth Resources Observation Science Center, 2017) |
| Human population density (number of people per km^2^) (year 2011-2019) | 1 km | 0.000007 to 134.45 | Anthropogenic | WorldPop (WorldPop and Center for International Earth Science Information Network (CIESIN) Columbia University, 2018) |
| Historical annual precipitation (mm) (year 2010-2018) | 1 km | 0 to 4285.3 | Climate | WorldClim (Fick and Hijmans, 2017) |
| Historical minimum temperature (°C) (year 2010-2018) | 1 km | 13.54 to 21.48 | Climate | WorldClim (Fick and Hijmans, 2017) |
| Historical maximum temperature (°C) (year 2010-2018) | 1 km | 21.83 to 32.25 | Climate | WorldClim (Fick and Hijmans, 2017) |
| Historical water vapor pressure (kPa) | 1 km |  | Climate | WorldClim (Fick and Hijmans, 2017) |
| Historical wind speed (m s^-1^) (year 1970-2000) | 1 km | 1.09 to 2.45 | Climate | WorldClim (Fick and Hijmans, 2017) |
| *Macaca nemestrina* occurrence probability | 5 km | 0 to 1 | Wildlife | (Moyes et al., 2016) |
| *Macaca fascicularis* occurrence probability | 5 km | 0 to 1 | Wildlife | (Moyes et al., 2016) |
| *Anopheles* Leucosphyrus group occurrence probability | 5 km | 0 to 1 | Wildlife | (Moyes et al., 2016) |
| Oil palm (year 2017) | 30 m | 0 (absence) and 1 (presence) | Landscape | (Danylo et al., 2021) |
| Mangrove (year 2000) | 30 m | 0 (absence) or 1 (presence) | Landscape | Socioeconomic Data and Application Centre (SEDAC) (Giri et al., 2005) |
| Peat lands (polygon data) | - | 0 (absence) or 1 (presence)* | Landscape | Global Forest Watch (Global Forest Watch, 2019) |
| Tree loss extent (year 2010-2019) | 30 m | 0 (absence) or 1 (presence) | Landscape | (Hansen et al., 2013) |
| Cropland extent (year 2015) | 30 m | 0 (absence) or 1 (presence) | Landscape | Geological Survey Global Food Security-Support Analysis Data (USGS GFSAD) (Thenkabail et al., 2021) |
| Forest/Non-forest extent | 25 m | 0 (absence) or 1 (presence) | Landscape | JAXA’s ALOS PALSAR-2 (Earth Observation Research Center, 2020) |
| Human Footprint index (year 2009) | 1 km | 0 to 50 | Anthropogenic | SEDAC (Venter et al., 2018) |
| Distance to open-water coastline (km) | 1 km | 0 to 49 | Landscape | WorldPop (WorldPop and Center for International Earth Science Information Network (CIESIN) Columbia University, 2018) |
| City with >10000 people (coordinate point data) | - |  | Anthropogenic | GeoNames (GeoNames, 2021) |

*After converted from polygon vector layer into raster layer

**Supplementary Table 2.** Classification of forest extent into dense forest and secondary forest.

| Level 1 classification | Level 2 classification | Description |
| --- | --- | --- |
| Forest | Dense forest | Primary/intact forest, old growth secondary forest, no human disturbance (eg. deforestation, agricultural land, etc), contiguous area of over 0.5 ha, ≥90% tree cover (Shimada et al., 2014). |
|  | Secondary forest | Fragmented forest, young forest, tree plantation, ≥60% tree cover |

**Supplementary Table 3.** Processed proximity map

| Processed proximity map layer | Source raster layer |
| --- | --- |
| Distance to forest | Forest extent |
| Distance to dense forest | Dense forest extent |
| Distance to secondary forest | Secondary forest extent |
| Distance to cropland | Cropland extent layer from USGS GFSAD |
| Distance to water bodies | Water bodies extent |
| Distance to urban area | Urban extent |
| Distance to tree loss extent | Tree loss extent |

**Supplementary Table 4.** Value range of each parameter involved in hyperparameter tuning for XGBoost modelling.

| **Parameters** | **Value range for hyperparameter tuning** | **Optimal value** |
| --- | --- | --- |
| Number of decision trees (nrounds) | 50 to 5000 | 150 |
| Maximum depth of a tree (max_depth) | 2 to 9 | 3 |
| Subsample ratio of columns when constructing each tree (colsample_bytree) | 0.1 to 1 | 0.9 |
| Step size shrinkage (eta) | 0.01 to 3 | 0.05 |
| Minimum loss reduction (gamma) | 0 to 10 | 0 |
| Minimum sum of instance weight (hessian) needed in a child (min_child_weight) | 1 to 10 | 1 |
| Subsample ratio of the training instances (subsample) | 0.1 to 1 | 0.7 |

**Supplementary Table 5.** The quantile-based classification and scoring of covariate and risk for development of priority zone map. Values within first and second quarters were assigned with score 0. Else, score 1 was assigned to values within third quarter and score 2 was assigned to values within fourth quarter.

| Criteria | Quarter | Values | Relative priority score |
| --- | --- | --- | --- |
| Risk map | 1 | ≤ 0.08 | 0 |
|  | 2 | > 0.08 and ≤ 0.19 | 0 |
|  | 3 | > 0.19 and ≤ 0.42 | 1 |
|  | 4 | > 0.42 | 2 |
| Agricultural and forest cover | 1 | ≤ 2% | 0 |
|  | 2 | > 2% and ≤ 55% | 0 |
|  | 3 | > 55% and ≤ 97% | 1 |
|  | 4 | > 97% | 2 |
| *Anopheles* Leucosphyrus group occurrence probability | 1 | ≤ 0.38 | 0 |
|  | 2 | > 0.38 and ≤ 0.49 | 0 |
|  | 3 | > 0.49 and ≤ 0.60 | 1 |
|  | 4 | > 0.60 | 2 |
| *M. fascicularis* occurrence probability | 1 | ≤ 0.19 | 0 |
|  | 2 | > 0.19 and ≤ 0.43 | 0 |
|  | 3 | > 0.43 and ≤ 0.69 | 1 |
|  | 4 | > 0.69 | 2 |
| *M. nemestrina* occurrence probability | 1 | ≤ 0.11 | 0 |
|  | 2 | > 0.11 and ≤ 0.29 | 0 |
|  | 3 | > 0.29 and ≤ 0.55 | 1 |
|  | 4 | > 0.55 | 2 |

**References**

Balk, D., Leyk, S., Jones, B., Montgomery, M. R., Clark, A. (2018). Understanding urbanization: a study of census and satellite-derived urban classes in the United States, 1990-2010. PLoS One. 13:e0208487. doi:10.1371/journal.pone.0208487

Buchhorn, M., Smets, B., Bertels, L., de Roo, B., Lesiv, M., Tsendbazar, N.-E., et al. Data from: Copernicus global land service: land cover 100m: collection 3: epoch 2019: globe. (2020) https://zenodo.org/record/3939050#.Yw-ZpXZBzIU

Danylo, O., Pirker, J., Lemoine, G., Ceccherini, G., See, L., McCallum, I., et al. (2021). A map of the extent and year of detection of oil palm plantations in Indonesia, Malaysia and Thailand. Scientific Data. doi:10.1038/s41597-021-00867-1

Earth Observation Research Center. Data from: JAXA global PALSAR-2/PALSAR/JERS-1 mosaic and forest/non-forest maps. Earth Observation Research Center. (2020) https://www.eorc.jaxa.jp/ALOS/en/palsar_fnf/data/index.html

Earth Resources Observation Science Center. Data from: Shuttle radar topography mission (SRTM) 1 arc-second global. United States Geological Survey. (2017) https://www.usgs.gov/centers/eros/science/usgs-eros-archive-digital-elevation-shuttle-radar-topography-mission-srtm-1-arc?qt-science_center_objects=0#qt-science_center_objects

Fick, S. E., Hijmans, R. J. (2017). WorldClim 2: new 1-km spatial resolution climate surfaces for global land areas. International Journal of Climatology. 37:4302-15. doi:10.1002/joc.5086

GeoNames. Data from: GeoNames gazetteer. GeoNames. (2021) https://download.geonames.org/export/dump/

Giri, C., Ochieng, E., Tieszen, L. L., Zhu, Z., Singh, A., Loveland, T., et al. Data from: Global mangrove forests distribution, 2000. NASA Socioeconomic Data and Applications Center (SEDAC). (2005) https://doi.org/10.7927/H4J67DW8

Global Forest Watch. Data from: Malaysia peat lands. Global Forest Watch. (2019) https://data.globalforestwatch.org/datasets/8d8462fca7b74b298598490b85d3bd44_9

Hansen, M. C., Potapov, P. V., Moore, R., Hancher, M., Turubanova, S. A., Tyukavina, A., et al. (2013). High-resolution global maps of 21st-century forest cover change. Science. 342:850-3. doi:10.1126/science.1244693

Loveland, T. R., Belward, A. S. (1997). The international geosphere biosphere programme data and information system global land cover data set (DISCover). Acta Astronautica. 41:681-9. doi:10.1016/S0094-5765(98)00050-2

Moyes, C. L., Shearer, F. M., Huang, Z., Wiebe, A., Gibson, H. S., Nijman, V., et al. (2016). Predicting the geographical distributions of the macaque hosts and mosquito vectors of *Plasmodium knowlesi* malaria in forested and non-forested areas. Parasit. Vectors. 9:242. doi:10.1186/s13071-016-1527-0

Shimada, M., Itoh, T., Motooka, T., Watanabe, M., Shiraishi, T., Thapa, R., et al. (2014). New global forest/non-forest maps from ALOS PALSAR data (2007–2010). Remote Sensing of Environment. 155:13-31. doi:10.1016/j.rse.2014.04.014

Thenkabail, P. S., Teluguntla, P. G., Xiong, J., Oliphant, A., Congalton, R. G., Ozdogan, M., et al. Global cropland-extent product at 30-m resolution (GCEP30) derived from Landsat satellite time-series data for the year 2015 using multiple machine-learning algorithms on Google Earth Engine cloud. Report. Reston, VA; 2021. Report No.: 1868.

Venter, O., Sanderson, E. W., Magrach, A., Allan, J. R., Beher, J., Jones, K. R., et al. Data from: Last of the wild project, version 3 (LWP-3): 2009 human footprint, 2018 release. NASA Socioeconomic Data and Applications Center (SEDAC). (2018) https://doi.org/10.7927/H46T0JQ4

WorldPop, Center for International Earth Science Information Network (CIESIN) Columbia University. Data from: Geospatial covariate data layer. WorldPop. (2018) https://www.worldpop.org/geodata/summary?id=23893

WorldPop, Center for International Earth Science Information Network (CIESIN) Columbia University. Data from: Individual countries 1km UN adjusted population density (2000-2020). WorldPop. (2018) https://www.worldpop.org/doi/10.5258/SOTON/WP00675
